# Growth and autolysis of the kefir yeast *Kluyveromyces marxianus* in lactate culture

**DOI:** 10.1038/s41598-021-94101-y

**Published:** 2021-07-15

**Authors:** Shou-Chen Lo, Chia-Yin Yang, Dony Chacko Mathew, Chieh-Chen Huang

**Affiliations:** 1 Department of Life Sciences, National Chung Hsing University, Taichung, 402 Taiwan; 2 Washington High School, Taichung, 406 Taiwan; 3 Program in Microbial Genomics, National Chung Hsing University, Taichung, 402 Taiwan; 4 Innovation and Development Center of Sustainable Agriculture, National Chung Hsing University, Taichung, 402 Taiwan

**Keywords:** Fungal biology, Fungal systems biology, Symbiosis, Proteome

## Abstract

*Kluyveromyces marxianus* is a yeast that could be identified from kefir and can use a broad range of substrates, such as glucose and lactate, as carbon sources. The lactate produced in kefir culture can be a substrate for *K. marxianus*. However, the complexity of the kefir microbiota makes the traits of *K. marxianus* difficult to study. In this research, we focused on *K. marxianus* cultured with lactate as the sole carbon source. The optimal growth and released protein in lactate culture were determined under different pH conditions, and the LC–MS/MS-identified proteins were associated with the tricarboxylic acid cycle, glycolysis pathway, and cellular stress responses in cells, indicating that autolysis of *K. marxianus* had occurred under the culture conditions. The abundant glyceraldehyde-3-phosphate dehydrogenase 1 (GAP1) was cocrystallized with other proteins in the cell-free fraction, and the low transcription level of the *GAP1* gene indicated that the protein abundance under autolysis conditions was dependent on protein stability. These results suggest that lactate induces the growth and autolysis of *K. marxianus*, releasing proteins and peptides. These findings can be fundamental for *K. marxianus* probiotic and kefir studies in the future.

## Introduction

*Kluyveromyces marxianus* is frequently isolated from dairy products, such as cheese and kefir. It is consistently detected in certain kefir cultures^1^ and can be acquired from unpasteurized milk^2^. The long history of safe use of these products makes *K. marxianus* a generally recognized as safe (GRAS) yeast strain. In a recent review, *K. marxianus* was considered as a potential probiotic yeast^3^. *K. marxianus* can utilize a broad spectrum of carbon sources, including carbohydrates and organic acids, such as glucose, sucrose, lactose, fructose, galactose, xylose, lactic acid and malic acid^4^, making it suitable for use in the food industry. In kefir cultures, lactate can be a nonfermentable carbon source for *K. marxianus*. However, the complexity of the symbiotic bacteria and yeasts in kefir culture makes the interactivity of the microbiota difficult to study. To simplify the interaction between *K. marxianus* and lactate, we focused on the traits of the *K. marxianus* lactate culture.

Although several nonfermentable carbon sources can be used to grow *K. marxianus*, protein expression under these culture conditions is rarely discussed. Another issue with using nonfermentable carbon sources as substrates is autolysis. The known autolysis in *Saccharomyces cerevisiae* occurs after fermentable carbon sources are exhausted at stationary phase in the culture^5^. Substitution of a fermentable carbon source with a nonfermentable carbon source can lead to the formation of a stressful environment for yeast.

Lactate is a nonfermentable carbon source for yeast. It is known that some *S. cerevisiae* strains can express pectinase in the presence of lactate^6^. The induction of secreted pectinase expression implies that lactate can upregulate the expression of certain proteins in *S. cerevisiae*. Furthermore, both *S. cerevisiae* and *K. marxianus* can reduce lactate feedback inhibition of lactic acid bacteria^7^. Therefore, with lactate as the sole carbon source, *K. marxianus* could exhibit potential for biotechnological applications. The lactate uptake mechanism of *K. marxianus* involves uptake of lactate anions with a monocarboxylate uniport^8^. Hence, the lactate uptake efficiency depends on the pH of the culture medium for the dissociation of lactate. Culture conditions with nonfermentable carbon sources and low pH cause autolysis in *S. cerevisiae* during winemaking^9^. Autolysis of yeast leads to the release of low-molecular-weight compounds and peptides that impart additional flavor or sweetness to the wine^9,10^. In this study, the *K. marxianus* strain Bot3 + 7 that was isolated from homemade kefir in Taiwan was classified by multilocus sequence typing^11^ and the proteins that were released from *K. marxianus* autolysis and the transcription level of the genes that encoded the high-abundance proteins were investigated. The total released proteins were analyzed by SDS-PAGE and liquid chromatography-tandem mass spectrometry (LC–MS/MS). The results showed that maximum protein release occurred in culture medium at pH 5.2, and the most abundant proteins were identified.

## Results

### Classification of *K. marxianus* Bot3 + 7 by multilocus sequence typing

To classify the isolated *K. marxianus* Bot3 + 7 strain, five *K. marxianus* housekeeping gene sequences, *IPP1*, *TFC1*, *GPH1*, *GSY2* and *SGA1*, were amplified by polymerase chain reaction according to previous publication^11^ and analyzed at (kmarxianusMLST.ucc.ie)^11^. The results indicate that *K. marxianus* Bot3 + 7 strain was most related to *K. marxianus* CCT 7735 strain which was isolated from regional Brazilian dairy industry wastewater^12^ (Supplementary Fig. S1).

### The initial pH value of the lactate culture medium affects the growth of *K. marxianus*

**Figure 1:**
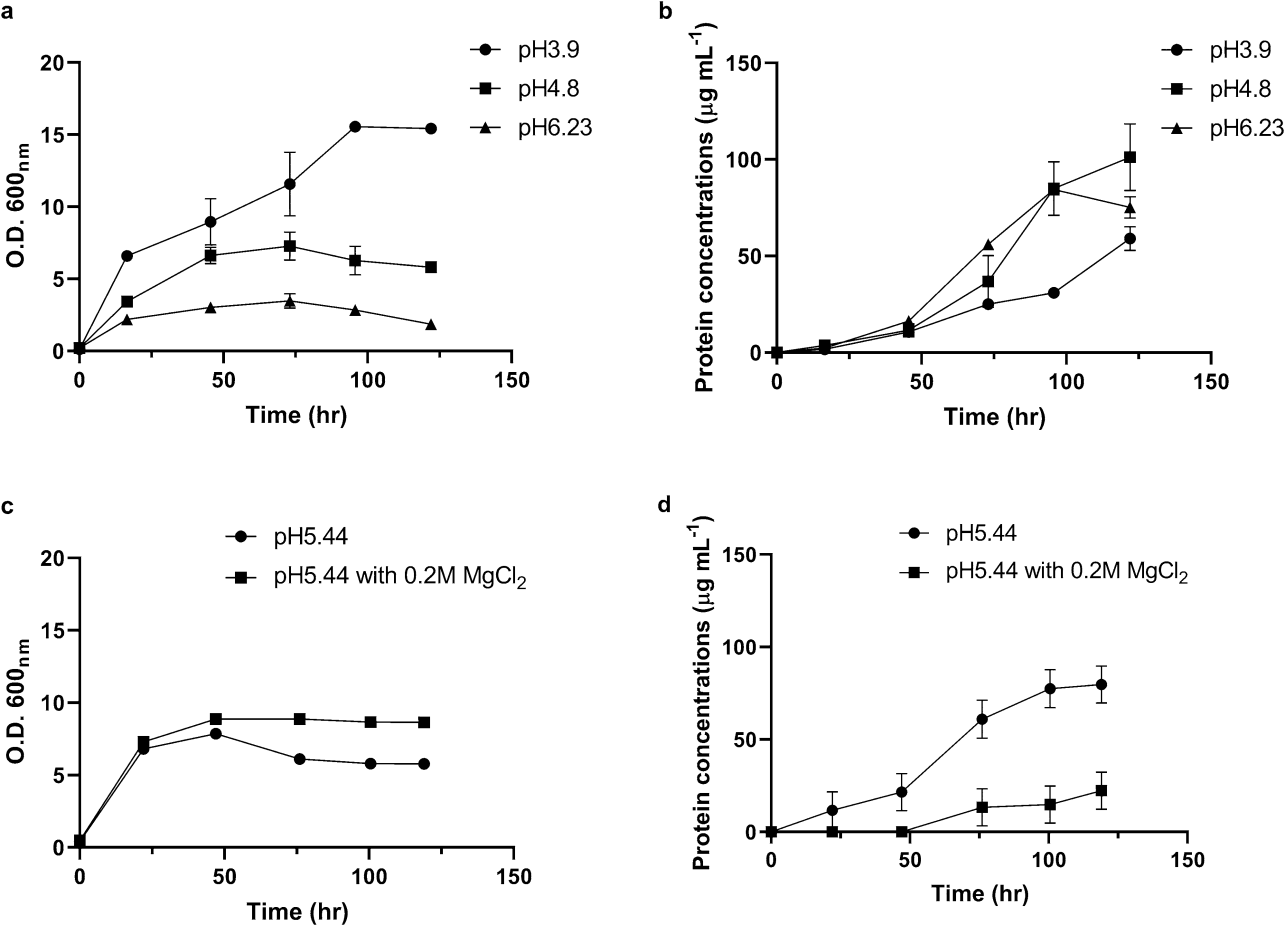
Growth curves and released protein concentrations of *K. marxianus* strain Bot3 + 7 at different pH values or with Mg supplementation. (**a**) Growth curve of *K. marxianus* strain Bot3 + 7 at pH 3.9, pH 4.8, and pH 6.23. (**b**) Released protein concentration of *K. marxianus* strain Bot3 + 7 at pH 3.9, pH 4.8, and pH 6.23. (**c**) Growth curve of *K. marxianus* strain Bot3 + 7 at pH 5.44 in the presence or absence of 0.2 M MgCl_2_. (**d**) Released protein concentration of *K. marxianus* strain Bot3 + 7 at pH 5.44 in the presence or absence of 0.2 M MgCl_2_. This image was created by using GraphPad Prism version 8.2.1 (https://www.graphpad.com/scientific-software/prism/).

The *K. marxianus* Bot3 + 7 strain was cultured in YPD medium for two days as a seed culture. Then, synthetic yeast culture medium with lactate as the sole carbon source, YNL, with different initial pH values was used for batch cultivation of the *K. marxianus* strain for five days. Although trace of YPD medium would be added into the synthetic medium culture, the results showed no difference from the culture with washed cell pellets under carbon source low concentration conditions (Supplementary Fig. S2). Therefore, the trace of YPD medium was not consider to affect the growth and autolysis in the experiments. On the other hand, the seed culture of yeast descended easily and made the inoculation hard to be consistent (Table 1). However, the values of OD_600_ increased at least above fivefold after 5 days culture, therefore the small variation of the initial values of OD_600_ should not affect the results. The growth curves and the released protein concentrations are shown in Fig. 1a,b. Optimal growth was observed at pH 4.18 (optical density at 600 nm (OD_600_) = 19) (Table 1). Upon cultivation with YNL medium at pH values higher than 4.8, the cell concentration of the *K. marxianus* strain decreased (Table 1). This might be because the dissociation of lactate anions decreased with increasing pH in the medium. The consumption of lactic acid would also increase pH values to about 7.6 during cultivation at 72 h (Supplementary Fig. S3a,b). However, the high pH values would not affect the cell viability in the first four days of culture (Supplementary Fig. S3c). To confirm if the *K. marxianus* strain could grow at pH 7.6, different carbon sources, such as glycerol, glucose and lactic acid, were used as carbon sources to culture the yeast strain (Supplementary Fig. S4). These results indicate that the cell viability of the yeast strain was not affected by pH values from 4.5 to 7.6 (Supplementary Figs. S3c, S4b).

**Table 1:** Growth of *Kluyveromyces marxianus* Bot3 + 7 and protein concentrations in different culture media.

Medium	Initial OD_600_	OD_600_ (5 days)	Protein concentration (μg/mL, 5 days)
YNL^a^ pH 3.9	0.124 ± 0.006^c^	15.429 ± 0.634	59.112 ± 10.694
YNL pH 4.18	0.583 ± 0.005	19.008 ± 0.025	99.394 ± 10.768
YNL pH 4.8	0.2 ± 0.0119	5.813 ± 0.192	101.228 ± 29.918
YNL pH 5.2	0.172 ± 0.007	6.864 ± 1.365	178.528 ± 50.534
YNL pH 5.44	0.412 ± 0.002	5.780 ± 0.020	79.685 ± 10.011
YNL pH 6.23	0.208 ± 0.005	1.850 ± 0.110	75.185 ± 9.579
YNL pH 5.44 + Mg^b^	0.482 ± 0.004	8.641 ± 0.097	22.290 ± 10.122

^a^YNL indicates synthetic medium with 2% lactic acid as a carbon source.

^b^0.2 M MgCl_2_ was present in the YNL medium.

^c^The data are expressed as the mean ± standard deviation; n = 3 independent biological samples.

### Protein released to the culture medium

Table 1 shows that the concentration of protein released to the culture medium was associated with the initial pH of the culture medium. Maximum protein release was observed at pH 5.2 (178 μg/mL) (Table 1). While using glycerol as carbon sources, the concentrations of protein released to the culture medium were no more than 10 μg/mL at pH 4.0, pH 4.9 and pH 6.3 (Supplementary Fig. S5). These results indicate that using lactic acid as carbon sources would induce some biological processes different from using glycerol as carbon sources. Despite the concentrations of released protein were different, it was hard to tell the differences of the cell morphology between these two culture conditions under microscope (Supplementary Fig. S6). According to Nambu-Nishida et al.’s report^13^, the divalent metal ion Mg^2+^ may affect many cellular and biological processes by changing the membrane permeability. In a previous study, the presence of MgCl_2_ decreased autolysis in *S. cerevisiae*^14^. Therefore, the presence of Mg^2+^ ions might help the *K. marxianus* strain grow with lactate and decrease autolysis. In the presence of 0.2 M MgCl_2_, the final cell concentration (OD_600_) at pH 5.44 increased from 5.78 to 8.64, and the released protein concentration decreased from 79.6 to 22.2 μg/mL (Fig. 1c,d, Table 1).

### Culture medium filtrate and protein concentration

**Figure 2:**
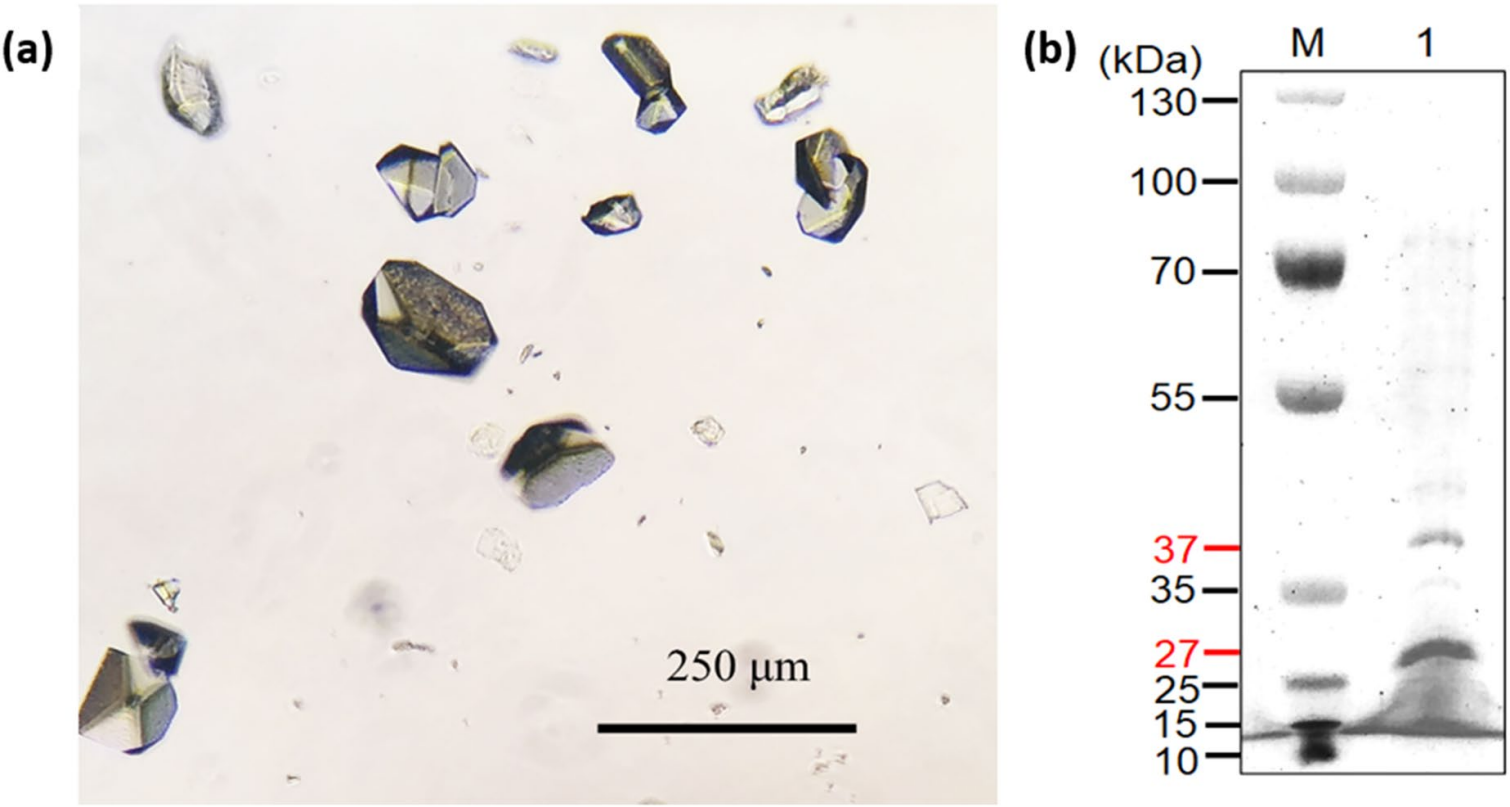
Microscopic photograph and SDS-PAGE analysis of protein crystals from the YNL culture filtrate at pH 5.44. (**a**) Microscopic photograph of the protein crystals. (**b**) SDS-PAGE analysis of the YNL culture filtrate at pH 5.44. Lanes M and 1 indicate the protein ladder and protein crystals, respectively. This image was edited by using Microsoft Office Professional 2019 PowerPoint (https://www.microsoft.com/zh-tw/microsoft-365/p/office-%E5%B0%88%E6%A5%AD%E7%89%88-2019/cfq7ttc0k7c5?activetab=pivot%3aoverviewtab).

**Figure 3:**
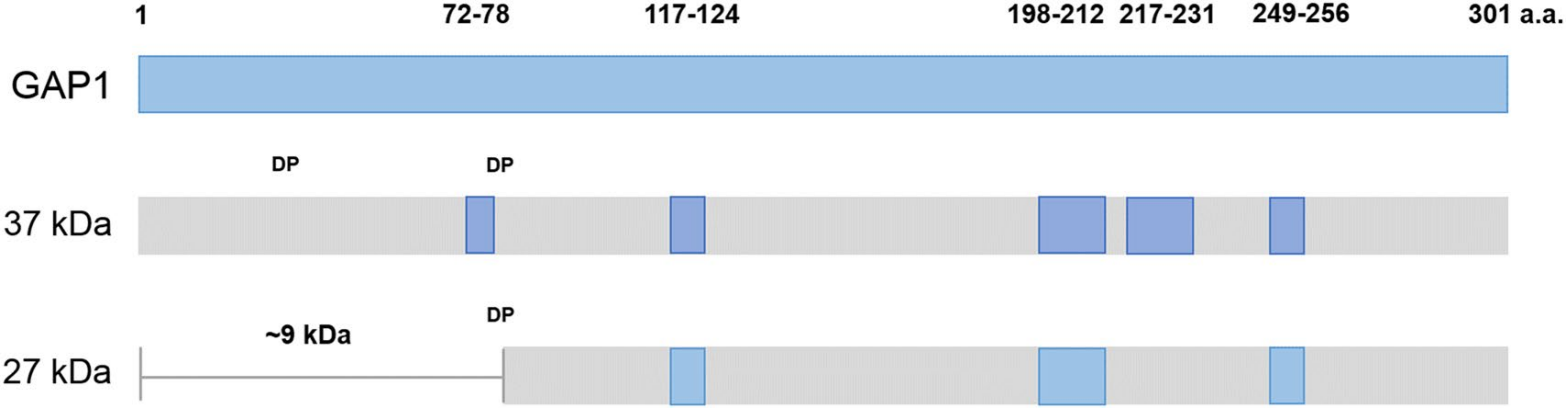
Schematic diagram of the peptides that matched to glyceraldehyde-3-phosphate dehydrogenase (GAP1) from the 37-kDa and 27-kDa protein bands in SDS-PAGE analysis. This image was created by using Microsoft Office Professional 2019 PowerPoint (https://www.microsoft.com/zh-tw/microsoft-365/p/office-%E5%B0%88%E6%A5%AD%E7%89%88-2019/cfq7ttc0k7c5?activetab=pivot%3aoverviewtab).

The released protein level in the culture medium was too low for direct analysis by SDS-PAGE or LC–MS/MS. The culture media were first filtered with membranes with a molecular weight cutoff of 3k as described in the “Materials and methods” section. The proteins were concentrated 20-fold for SDS-PAGE analysis (Supplementary Fig. S7). However, the amount of protein was not suitable for total protein LC–MS/MS analysis. In order to have more reliable results, the condition with small variation of protein concentration was used in the experiment. Therefore, 300 mL of culture medium (at pH 5.44 instead of pH 5.2) in another batch experiment of *K. marxianus* Bot3 + 7 was collected to obtain more protein. Approximately 0.1 mg/mL protein was obtained on the 12th day (288 h) of incubation (data not shown). It took more than twice as much time to reach the optimum concentration of released protein than it did with the 3-mL culture (Fig. 1b). The medium was collected at 120 h and 288 h, filtered through a 0.2-μm pore size membrane and stored at 4 °C for further concentration and desalting. Noticeable small crystals appeared in the storage medium of the 288-h sample after 2 days (Fig. 2a). The crystals formed in the culture medium did not dissolve in ddH_2_O and methanol but could be dissolved in Bradford reagent and low-pH solutions, such as 10% acetic acid. The crystals dissolved in Bradford reagent were analyzed by SDS-PAGE, and there were two major bands at approximately 37 kDa and 27 kDa (Fig. 2b) along with other faint bands. To determine the identity of the proteins, the major bands were excised and sent for LC–MS/MS analysis. Peptide fingerprinting of the 37-kDa and 27-kDa proteins identified both proteins as glyceraldehyde 3-phosphate dehydrogenase 1 (GAP1) in *K. marxianus* (Fig. 3). Some protein regions were unmatched, which might have been due to insufficient in-gel trypsin digestion. Nonetheless, a notable fingerprint peptide (VAVFQEK) was absent just before the second acid cleavage site in the peptide (aspartic acid-proline, 79D-80P) among the 27-kDa peptide fingerprints compared with the 37-kDa fingerprints (Fig. 3, Supplementary Table S1). The molecular mass of the N-terminal peptides (residues 1–79) after acid cleavage of G3P1 was approximately 9 kDa. These results suggest that the 27-kDa protein was derived from the acid cleavage of GAP1 when the protein was dissolved in acidic solutions.

### General proteomics of released protein from *K. marxianus* in lactate culture medium

#### Analysis of total released protein with LC–MS/MS

Nonetheless, protein crystals were formed, and the proteins in YNL medium at pH 5.44 were further desalted and concentrated to at least 3 μg/μL for LC–MS/MS analysis as described in the “Materials and methods” section. Note that since the samples were released by autolysis, the proteins would be cleaved at unexpected cleavage sites. To maintain reliability of the LC–MS/MS analysis, only trypsin cleavage-derived peptides were used as fingerprints to identify proteins. Therefore, the numbers of matched peptides were lower than expected. However, the identified proteins could be confirmed in the autolysis medium. A total of 313 and 513 proteins were matched in the NCBI *K. marxianus* protein database in the 120-h and 288-h culture media, respectively. However, only 295 and 473 of the identified proteins in the 120-h and 288-h culture media, respectively, could be mapped to UniProtKB for further Gene Ontology (GO) classification. Generally, the mapped proteins in the 120-h medium were similar to those in the 288-h medium, but some of the proteins were missing, such as actin, which could be found in only the 120-h cell pellets and the 288-h medium, not in the 120-h medium (Supplementary Tables S2–S4). This was probably because the autolysis process was closer to completion in the 288-h samples than in the 120-h samples.

#### Metabolism

Proteins associated with the tricarboxylic acid cycle, gluconeogenesis, and pentose phosphate pathway were identified (Supplementary Tables S3, S4). Among other carbohydrate metabolism processes, proteins related to galactose metabolism and exoinulinase were identified (Supplementary Tables S3, S4). Two proteins were identified as being related to lactate metabolism (D-lactate dehydrogenase [cytochrome] 2 and cytochrome b2) (Supplementary Tables S3, S4).

#### Stress response

The identified proteins included several proteins that were related to cellular stress responses, such as responses to starvation (4 proteins in the 120-h medium, 5 proteins in the 288-h medium), osmotic stress (3 proteins in the 120-h medium, 8 proteins in the 288-h medium), and oxidative stress (16 proteins in the 120-h medium, 17 proteins in the 288-h medium). Notably, a 12 kDa heat shock protein (HSP12) was related to both osmotic and oxidative stress; therefore, it could be counted as only 1 protein, making the total 22 in the 120-h medium and 29 in the 288-h medium. A total of 11 and 13 proteins (including heat shock proteins and chaperones) in the 120-h and 288-h media, respectively, that help in protein folding and refolding were identified in the samples (Supplementary Tables S3, S4).

### Abundance of identified proteins

The protein abundance is presented as a percentage of the exponentially modified protein abundance index (emPAI%)^15^, and the 15 most abundant proteins (emPAI% > 0.5) in the 288-h medium are listed in Table 2. Note that several proteins, such as enolase and triosephosphate isomerase, had a higher emPAI% in the 120-h medium than in the 288-h medium. This indicates that the released proteins were degraded during autolysis. Therefore, these 15 most abundant proteins in Table 2 were relatively stable proteins in the experiments. The major groups of proteins were related to metabolic pathways and the heat shock response. GAP1, enolase (ENO), phosphoglycerate kinase (PGK), phosphoglycerate mutase 1 (GPM1), fructose-bisphosphate aldolase (FBA1) and pyruvate decarboxylase (PDC1) were related to the gluconeogenesis pathway. Heat shock proteins, including HSP12, heat shock protein 26 (HSP26), and heat shock protein SSA3 (SSA3), were associated with the response to stress conditions. The other proteins were triosephosphate isomerase (TPI1), transaldolase (TAL1), alcohol dehydrogenase 2 (ADH2), alcohol dehydrogenase (adh), elongation factor 2 (EFT1) and D-arabinose dehydrogenase [NAD (P)+] heavy chain (ARA1) (Table 2). The most abundant protein, HSP12, had a notable abundance ratio compared to all the other matched proteins (Table 2). However, GAP1 cocrystallized during storage before LC–MS/MS analysis, so the abundance ratio of GAP1 in the total released proteins was underestimated.

**Table 2:** The 15 most abundant proteins in the 288-h samples from LC–MS/MS analysis.

NCBI accession number	Protein name	*M*_*r*_	120 h pellet			120 h medium			288 h medium		
			Matches	Sequences	emPAI%^a^	Matches	Sequences	emPAI%	Matches	Sequences	emPAI%
BAO39229.1	12-kDa heat shock protein	11,193	34 (28)	8 (7)	5.68	40 (37)	9 (8)	4.76	54 (52)	10 (9)	18.7
P84998.1	Glyceraldehyde-3-phosphate dehydrogenase 1	35,231	76 (70)	17 (17)	2.32	55 (46)	15 (14)	0.47	99 (90)	20 (19)	3.51
BAO38084.1	Enolase	46,773	63 (55)	20 (19)	0.9	168 (141)	30 (26)	2.86	128 (115)	20 (19)	2.3
BAO40149.1	Triosephosphate isomerase	26,979	29 (25)	13 (11)	0.88	93 (84)	23 (22)	4.65	48 (45)	15 (14)	1.43
BAO38162.1	Phosphoglycerate kinase	44,418	25 (23)	13 (13)	0.32	135 (123)	29 (28)	1.66	84 (76)	19 (19)	1.35
BAO39929.1	Transaldolase	36,452	33 (29)	15 (15)	0.71	102 (87)	28 (25)	2.32	41 (37)	18 (18)	1.21
BAP70103.1	Phosphoglycerate mutase 1	27,480	36 (31)	11 (11)	1.18	63 (52)	14 (13)	1.28	44 (42)	12 (11)	1.16
BAO42678.1	Heat shock protein SSA3	70,036	46 (39)	21 (20)	0.4	119 (108)	29 (29)	0.68	93 (86)	27 (26)	0.81
BAO40152.1	Heat shock protein 26	21,932	25 (24)	6 (6)	0.35	22 (21)	6 (6)	0.25	49 (46)	7 (7)	0.7
Q9P4C2.3	Alcohol dehydrogenase 2	36,945	21 (15)	12 (11)	0.32	27 (23)	14 (13)	0.29	42 (38)	16 (15)	0.67
BAO40412.1	Fructose-bisphosphate aldolase	39,472	27 (25)	12 (11)	0.25	53 (49)	17 (16)	0.43	57 (53)	14 (14)	0.62
QGN17490.1	Pyruvate decarboxylase	61,862	48 (42)	19 (18)	0.41	67 (57)	20 (18)	0.42	68 (61)	20 (19)	0.6
CAA42785.1	Alcohol dehydrogenase	41,661	54 (47)	14 (12)	0.51	31 (27)	12 (12)	0.26	55 (52)	11 (11)	0.59
BAO39040.1	Elongation factor 2	93,293	42 (33)	20 (17)	0.17	71 (61)	27 (25)	0.18	108 (95)	33 (31)	0.56
BAO39872.1	d-arabinose dehydrogenase [NAD (P)+] heavy chain	38,883	10 (8)	8 (8)	0.15	9 (7)	7 (5)	0.05	46 (43)	13 (13)	0.55

^a^The emPAI% is presented as the mean of at least three replicates.

### Potential strong promoters under lactate culture conditions

**Figure 4:**
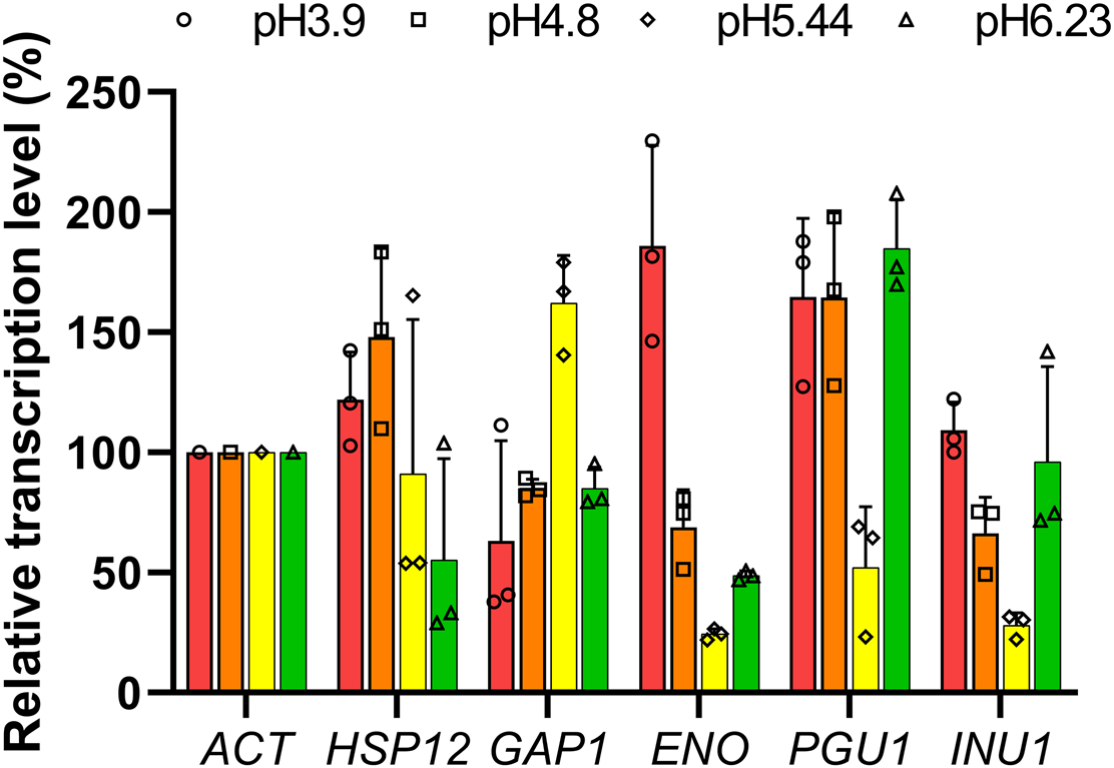
Relative transcription levels of *HSP12*, *GAP1*, *ENO*, *PGU1*, and *INU1* compared with those of *ACT* at pH 3.9, pH 4.8, pH 5.44, and pH 6.23 in culture. The data are presented as the mean and standard deviation of three replicates. This image was created by using GraphPad Prism (version 8.2.1; https://www.graphpad.com/scientific-software/prism/).

High abundance of proteins in culture medium may be a result of pH-induced expression, protein stability, or both. To further investigate the transcription levels of the matched protein-encoding genes, the expression of the *HSP12*, *GAP1*, and *ENO* genes was measured by using quantitative real-time polymerase chain reaction (qPCR) after 120 h of culture (Fig. 4). In addition, the potential promoters of *INU1* and *PGU1*, which encode inulinase and polygalacturonase, respectively, were also analyzed. The promoter of *INU1* was applied for heterologous protein expression^16,17^, and the promoter of *PGU1* has potential biotechnological applications in *K. marxianus*^18^. The transcription levels of all the genes were determined by qPCR and compared with that of the actin (*ACT*) gene to calculate the relative transcription levels. *HSP12* presented relatively high transcription levels at pH 3.9 and pH 4.8, and the transcription level decreased at pH 5.44 and pH 6.23 (Fig. 4). The *GAP1* transcription level was lower than that of *ACT* under all the tested conditions (Fig. 4). *ENO* transcription was high at pH 3.9 but decreased at pH 4.8, pH 5.44 and pH 6.23 (Fig. 4). The notably high transcription levels of *PGU1* at every tested pH except at pH 5.44 indicated the potential for application in heterologous enzyme expression. On the other hand, the transcription levels of *INU1* were lower than those of *PGU1* except at pH 5.44 (Fig. 4).

## Discussion

*Kluyveromyces marxianus* exhibits various phenotypes among strains, therefore it is important to classify the newly isolated strain at least on the genetic level for further study^11^. The strain most closely related to *K. marxianus* Bot3 + 7 was identified as *K. marxianus* CCT 7735 (Supplementary Fig. S1). Both strains were isolated from dairy sources and may have similar phenotypes, as discovered in this study. Lactate is the primary acid that is produced in kefir culture, and its level can range from 0.6 to 1% (w/v)^19,20^. On the other hand, application of the lactate assimilation trait of *K. marxianus* was reported to reduce the l-lactate feedback inhibition of *Lactococcus lactis* for nisin production^7^. This indicates that *K. marxianus* could play the same role during kefir production and the potential of lactate usage for biotechnological applications. In this study, we focused on using lactate as the sole carbon source to culture *K. marxianus*. The proteins released from *K. marxianus* under lactate culture conditions were identified.

DL-lactate was used as the sole carbon substrate for batch cultivation of *K. marxianus* at different pH values. Even under the optimal growth conditions at pH 4.18, as shown in the results (Table 1), the released protein concentrations and the SDS-PAGE analysis of released protein indicated that some of the yeast cells were still autolyzed (Supplementary Fig. S7). The mechanism of autolysis in *K. marxianus* is similar to the mechanism in *S. cerevisiae*^21,22^, which is based on the biosynthesis of lytic enzymes, and optimum autolysis was observed at pH 4.5 in culture^22^. In this study, the optimum culture pH for autolysis was observed to be pH 5.2 (Table 1). However, the culture conditions were different from those in this study^22^, since this work lacked fermentable carbon sources in the culture medium. In the presence of 0.2 M MgCl_2_, the final cell concentrations and the released protein concentrations in the culture medium increased and decreased, respectively (Fig. 1c,d). The presence of excess Mg^2+^ ions facilitates protein secretion in *K. marxianus* (0.2 M MgSO_4_)^13^ and decreases autolysis in marine bacteria (0.5 M MgCl_2_) and *S. cerevisiae* (0.1% MgCl_2_)^14,23^. Although the mechanism remains unclear, it was proposed that Mg^2+^ ions might help organize cell membranes in both eukaryotes and prokaryotes^13^. The GAP1 and HSP12 proteins found in the culture filtrate were also related to the maintenance of cell membrane integrity and stability^24,25^. These results suggest that *K. marxianus* may bear the stress that could decompose membranes. Nonetheless, the inhibition of autolysis suggested that the presence of 0.2 M MgCl_2_ helped *K. marxianus* resist the stress occurring under the lactate culture conditions (Fig. 1c,d).

LC–MS/MS analysis of the total protein content revealed the identities of the proteins released during and after autolysis, other than the cocrystallized GAP1, and it also provided a glimpse of the metabolic pathways in *K. marxianus*. Enzymes in central metabolic pathways, such as the tricarboxylic acid cycle, glycolysis/gluconeogenesis, and pentose phosphate pathway, were identified (Supplementary Tables S2–S4). Although dl-lactate was the only carbon source that was supplied to the culture medium, proteins related to galactose and inulin metabolism were also expressed (Supplementary Tables S2–S4). These results suggest that *K. marxianus* has the potential to assimilate galactose and inulin under these culture conditions. Four lactate metabolism-related proteins, namely, carboxylic acid transporter, d-lactate dehydrogenase, d-lactate dehydrogenase 2 and cytochrome b2, were identified (Supplementary Table S2). d-lactate dehydrogenase [cytochrome] catalyzes the conversion of d-lactate to pyruvate with the reduction of two molecules of cytochrome. The association between these identified lactate assimilation proteins and the expression of the central metabolism enzymes indicates the lactate assimilation pathway in *K. marxianus*. It was reported that the *PGU1* promoter is activated in the presence of lactate in *S. cerevisiae*^6^. In the lactate culture of *K. marxianus*, the transcription level of *PGU1* was higher than that of *GAP1* but lower than that of *INU1* at pH 5.44 (Fig. 4). However, none of the analyzed peptides matched the PGU1 protein in our results. Among the 15 most abundant proteins in the 288-h culture medium, the average emPAI% of HSP12 was 18.7%, and that of the second abundant protein, GAP1, was 3.51% (Table 2). There was a 15.19% difference between these two proteins, and the third most abundant protein differed from GAP1 by only 1.21% (Table 2). These results suggest that most of the proteins were digested to unidentified peptides under the autolysis conditions. Therefore, PGU1 might be digested to unidentified peptides. However, none of the peptides matched PGU1, even though no enzyme digest fingerprints were considered (data not shown), which needs to be further investigated.

GAP1 was cocrystallized in the cell-free fraction and was found to be a relatively high abundance protein in the results at pH 5.44 in the 288-h culture medium (3.51 emPAI%) and 120-h pellets (2.32 emPAI%) (Table 2). On the other hand, actin exhibited only 0.191 emPAI% in the 120-h pellets (Supplementary Dataset). However, the transcription level of *GAP1* was lower than that of *ACT* (Fig. 4). These results suggest that the protein abundance under the autolysis condition was dependent on the stability of the protein, not on the gene transcription level. The most abundant protein, HSP12, was reported to increase membrane stability under different stress conditions in *S. cerevisiae*, such as heat shock and oxidative and osmotic stresses^25^. The expression of HSP12 in *S. cerevisiae* may also contribute to lifespan extension by protecting the membrane from desiccation^26,27^. The relatively high expression of *HSP12* (Fig. 4) under the autolysis conditions suggests that HSP12 might play a similar role in protecting *K. marxianus* cells.

Protein abundance was related to protein stability and to *K. marxianus* abundance under the autolysis conditions. However, one cannot exclude the possibility that proteins might also be secreted into the medium rather than released from dead yeast cells. For example, GAP1, enolase and pyruvate decarboxylase are secreted into the extracellular space in *Candida albicans* without N-terminal signal peptides^28^. These nonconventional protein secretion pathways were also found in *S. cerevisiae*^29^ and are related to nonclassical export (*NCE*) genes^30^ such as *NCE102*, which is also encoded in the *K. marxianus* genome^31^. In our results, while most of the GAP1 was in the cell pellet, most of the enolase was already in the culture medium at 120 h (Table 2). These findings suggest that nonconventional protein secretion occurred under lactate culture conditions. Therefore, the secreted proteins might also be digested to peptides as the rest of the proteins are released by autolysis and become substrates for other symbiosis-related proteins in kefir. The well-known symbiotic interaction between *Lactobacillus bulgaricus* and *Streptococcus thermophilus* in yogurt is that *S. thermophilus* produces formate to stimulate *L. bulgaricus*, and *L. bulgaricus* liberates free amino acids and peptides from milk proteins to stimulate *S. thermophilus *^32^. In this study, lactate was used as a substrate for *K. marxianus* growth and induced autolysis of *K. marxianus* to release proteins and peptides into the cell-free fraction. This suggests that the lactate produced by lactic acid bacteria would induce the release of proteins and peptides from *K. marxianus*. However, the functions of these proteins and peptides remain to be discovered by coculturing *K. marxianus* with lactic acid bacteria.

*Kluyveromyces marxianus* has received recognition for its potential industrial applications in recent years due to its thermotolerance, rapid growth rates, and broad substrate spectrum^33^. Although knowledge of its biochemistry and genetics is limited compared to that for *S. cerevisiae*, many studies based on different biochemical principles, such as nonhomologous end joining^34^, homologous recombination^35,36^, and the CRISPR/Cas9 mechanism^37^, have been applied to develop genetic engineering tools. These studies used fermentable substrates for culture, such as glucose and galactose. Therefore, knowledge on culturing nonfermentable substrates is limited. In this study, the optimal lactate culture conditions, the released proteins and the transcription levels of several genes were determined in *K. marxianus*. Autolysis occurred in all of the experimental lactate culture conditions, and the LC–MS/MS results including various heat shock proteins could be the foundation for *K. marxianus* probiotic and kefir research.

## Materials and methods

### Yeast strain and culture conditions

The *K. marxianus* Bot3 + 7 strain was isolated from homemade kefir in Taiwan. The yeast was first precultured for 2 days at 30 °C with rotation at 120 rpm in YPD medium containing 10 g of yeast extract, 20 g of peptone, and 20 g of glucose per liter of distilled water. To measure growth and protein release, precultured cells from a two-day culture were diluted 1:100 by adding 30 μL of the suspension to 3 mL of YNL medium (a synthetic medium composed of 6.7 g of yeast nitrogen-base without amino acids (Difco, Detroit, MI, USA) and 20 g of lactic acid (J.T. Baker, Philipsburg, NJ, USA) per liter of distilled water) or YNGlycerol medium (a synthetic medium composed of 6.7 g of yeast nitrogen-base without amino acids (Difco, Detroit, MI, USA) and 16 g of glycerol (Union Chemical Works, Hsinchu City, Taiwan) per liter of distilled water). The pH of the medium was adjusted by the addition of NaOH (Showa, Tokyo, Japan). All yeast cultures were incubated at 30 °C in a rotating incubator (120 rpm). The medium was supplemented with MgCl_2_ as indicated in the manuscript. To determine cell viability, the colony-forming unit (CFU) method was used. The yeast cells in culture medium were suspended in sterile water and diluted to a final concentration of about 10^3^ cells/mL. A 150 μL sample of the suspension was inoculated on solid YPD medium and incubated at 37 °C. The CFUs were counted after 48 h of incubation. For LC–MS/MS analysis of the total protein content, the precultured cells were diluted by adding 3 mL of the suspension to 300 mL of YNL medium (pH 5.44) and incubated at 30 °C in a shaking incubator (120 rpm) for LC–MS/MS protein analysis.

### Multilocus sequence typing

The genomic DNA of yeast was extracted with the FavorPrep Fungi/Yeast Genomic DNA Extraction Mini Kit (Favorgen, Taiwan) according to the manufacturer’s instructions. The DNA yield was determined by a Nano-100 Micro-Spectrophotometer (Medclub Scientific Co., Ltd., Taiwan). A total of 10 ng of genomic DNA was used in a 25 μL polymerase chain reaction (Phusion High-Fidelity DNA Polymerase, New England Biolabs, USA). The PCR conditions were as follows: initial denaturation at 98 °C for 50 s; followed by 35 cycles of denaturation at 98 °C for 30 s, annealing at 62 °C for 30 s and elongation at 72 °C for 1 min; followed by a final extension step at 72 °C for 7 min. The amplified DNA products were separated in a 0.8% (w/v) agarose gel in 0.5× TAE buffer (20 mM Tris, 10 mM acetic acid, 0.6 mM EDTA) at 50 V for 1 h, stained with SafeView (BioPioneer, Taiwan) and visualized with a LED transilluminator (BV 200, Clinx Science Instruments, China). The DNA bands were excised from the gels and sequenced in each direction by Genomics (Taiwan). The sequences were prepared as a multi fasta file by following the instructions on the *K. marxianus* MLST analysis website^11^. The phylogenetic tree was generated with the Interactive Tree Of Life online tool (version 6.0) under free access mode (itol.embl.de)^38^. All the primers used for analysis in this study are listed in Supplementary Table S5.

### Growth curves and protein concentration determination

The growth of the *K. marxianus* Bot3 + 7 strain was determined by measuring the OD_600_ and OD_595_ with a GeneQuant 1300 system (GE Healthcare, Little Chalfont, Buckinghamshire, UK) and a Tecan Sunrise Basic microplate reader (Tecan Austria GmbH, Grödig, Austria), respectively. At each time point during the 5-day cultivation period, 20 μL of yeast culture was sampled and aliquoted into a 96-well microplate in triplicate and diluted 1:10 by adding 180 μL of distilled water to measure the growth. Additionally, protein secretion into the external medium was determined by measuring the OD_595_ with a Tecan Sunrise Basic microplate reader using the Bradford method with bovine serum albumin as the standard by following the manufacturer's instructions (Bio-Rad Protein Assay Dye Reagent, Bio-Rad, Hercules, CA, USA). For this, 50 μL of yeast culture at each time point was centrifuged at 14,000 rpm for 3 min. After centrifugation, 40 μL of supernatant was sampled into a 96-well microplate in triplicate, and 160 μL of Bradford reagent (1.25-fold) was added to be diluted fivefold with the protein.

### High performance liquid chromatography (HPLC) analysis

The culture media of the samples were treated with 10% trichloroacetic acid and incubated on ice for 30 min. The supernatants were collected by centrifugation at 14,000 rpm at 4 °C for 15 min and followed by HPLC analysis. The concentrations of lactic acid and glycerol were determined by KNAUER PLATINblue UHPLC System (KNAUER, Berlin, Germany) equipped with an HPLC column Shodex SH1011 (particle size 6 μm, 300 mm × 8 mm, Showa Denko America, New York, NY, USA) and a refractive index (RI) detector (AZURA RID 2.1L, KNAUER, Berlin, Germany). The mobile phase was 5 mM H_2_SO_4_, and the flow rate was 0.6 mL/min. The column temperature was at 60 °C. The injection volume was 10 μL for each analysis.

### SDS-PAGE

The crystals were collected into 1.5-mL centrifuge tubes with pipet tips and washed with ddH_2_O. Then, the crystals were added and dissolved into Bradford reagent solutions until saturation. Ten microliters of sample and prestained protein marker (PageRuler Plus Prestained Protein Ladder, 10 to 250 kDa, Thermo Scientific) were used for SDS-PAGE analysis.

### Sample preparation for LC–MS/MS protein analysis

#### Proteins in the SDS-PAGE gel

The protein bands were first excised as small pieces from the gel. Then, 25 mM ammonium bicarbonate (ABC, Fluka) (dissolved in 50% acetonitrile (ACN, J.T. Baker, Philipsburg, NJ, USA)) was added. The mixture was incubated for 30 min at room temperature, which was repeated if the gel pieces were not destained. Dehydration was performed by the addition of 100% ACN. Reduction and alkylation were performed with the following steps: 10 mM dithiothreitol (DTT, Sigma) (dissolved in 50 mM ABC) was added to the gel pieces, followed by incubation for 60 min at 56 °C. The solutions were discarded, 55 mM iodoacetamide (IAA, dissolved in 50 mM ABC; Sigma) was added, and the gel pieces were incubated for 45 min in the dark at room temperature. The solutions were discarded, and the gel pieces were dehydrated with 100% ACN. Trypsin (12.5 ng/μL, dissolved in 50 mM ABC; Trypsin Gold, Promega) was added to the gel pieces, which were rehydrated at 4 °C for 30–60 min and then digested overnight at 37 °C. To recover the peptides from the gel, first, 30–100 μL of 5% formic acid (FA, dissolved in 50% ACN; Fluka) was added, and the gel pieces were agitated at room temperature for 30–60 min and transferred to a new centrifuge tube. Second, 15–50 μL of 5% FA was added, and the tube was incubated at room temperature for 10 min, followed by the addition of 15–50 μL of 100% ACN and agitation for 30–60 min. Third, the two extract solutions were combined and dried in a Speed Vac. The samples were desalted using C18 ZipTips (Millipore) with the following steps: the dried peptide mixtures were desalted in 10 μL of ddH_2_O containing 0.1% FA, a ZipTip was activated by rinsing 3 times with 10 μL of 100% ACN, the tip was equilibrated 3 times with 10 μL of 0.1% FA, peptides were bound to the tip by aspirating and dispensing the peptide solutions 10 times, the tip was washed 6 times with 10 μL of 0.1% FA, and peptides were eluted with 10 μL of 0.1% FA (dissolved in 50% ACN) by aspirating and dispensing 10 times. These steps were repeated to obtain a combined solution, and the combined solution was dried with a SpeedVac vacuum concentrator.

#### Proteins in cell pellets and autolysis medium

The cell pellets of the 120-h samples were collected and washed eight times with 40 mL of 50 mM Tris–HCl (pH 8.03) at 16 °C. The washed pellets were dissolved in 1 mL of 50 mM Tris–HCl (pH 8.03), and approximately 300 μL of 0.5 mm glass beads was added. The glass beads and pellet mixture were vortexed in a cyclomixer 30 times at 2500 rpm for 30 s each, immersing the cell suspension in ice for 30 s between vortexing cycles to obtain at least 3 μg/μL protein. The proteins in the supernatant of the 120-h and 288-h *K. marxianus* cultures were concentrated to at least 3 μg/μL using Amicon Ultra-15 3 k molecular weight cutoff membranes (Millipore) according to the manufacturer’s instructions. The autolysis medium samples were also dialyzed 3.7 × 10^–15^ times with 50 mM Tris–HCl (pH 8.03). Then, 1 μL of DTT (1 M) and 2.5 μL of 10% SDS were added to 20 μL of the samples, and the samples were cooled to room temperature after incubation for 10 min at 95 °C. Three microliters of 1 M IAA was added, and the samples were reacted for 30 min in the dark at room temperature. Twelve microliters of 40% acrylamide, 4.5 μL of 10% SDS, 1.5 μL of 10% APS and 0.5 μL of TEMED were added, and the samples were incubated for 30 min at room temperature to form a tube gel. The solutions were discarded, and 500 μL of ddH_2_O was added. Then, the gel was incubated for 30 min at room temperature and cut into small pieces. The small pieces were washed three times. First, 100 mM ABC was used for 15 min. Then, 50 mM ABC and 50% ACN were used for 30 min, followed by washing with 100% ACN for 10 min. After washing, the samples were dried with a SpeedVac vacuum concentrator. Trypsin (0.5 μg) was added, and the samples were digested at 37 °C for 16 h. ACN (100%) was added to the peptide extract and reacted for 30 min at room temperature, and then, the supernatant was transferred to a new tube. ACN (50%) and FA (1%) were added, and the samples were incubated for 60 min at room temperature. Then, the supernatant was transferred to a new tube. Subsequently, 100% ACN was added, and the samples were incubated for 10 min at room temperature. Then, the supernatant was transferred to a new tube. The supernatant was dried with a SpeedVac vacuum concentrator and resuspended in 20 μL of 0.1% FA (in ddH_2_O). The samples were desalted and concentrated using C18 ZipTip as mentioned previously. The samples were then resuspended in 50 μL of 0.1% FA (in ddH_2_O).

### LC–MS/MS protein analysis

LC–MS/MS analysis was performed on an UltiMate 3000 RSLCnano LC system (Thermo Fisher Scientific) coupled with a TripleTOF 6600 mass spectrometer (Applied Biosystems Sciex) equipped with information-dependent acquisition (IDA) mode and an electrospray ionization (ESI) source operating in positive mode.

#### Protein identification

The proteins in the SDS-PAGE gel were identified by using the following methods. The liquid chromatography conditions were as follows: separation of eluted peptides was performed on an Acclaim PepMap C18 analytical column (75 μm I.D. × 25 cm nanoViper, 2 μm particle size, 100 Å pore size, Thermo Fisher Scientific). Mobile phase A was 0.1% FA in ddH_2_O, and mobile phase B was 0.1% FA in 100% ACN. The gradient was as follows: 0–4.5 min 95% A, 4.5–31 min 95–65% A, 31–32 min 65–10% A, 32–52 min 10% A, 52–53 min 10–95% A, and 53–70 min 95% A. The flow rate was 300 nL/min at 35 °C, and the sample loading volume was 10 μL (0.1% FA). The samples were examined in triplicate.

The mass spectrometric conditions were as follows: The operating parameters of ESI mode were utilized: ion spray voltage + 2500 V, interface heater temperature 150 °C, declustering potential 80 V and column oven temperature 35 °C. The sheath gas and curtain gas were both nitrogen, and the pressures were 15 and 30 psi, respectively. The IDA mode was utilized. The TOF–MS scan range was set to 350–1250 m/z acclimated for 0.25 s, and the MS/MS scan range was set to 65–1800 m/z acclimated for 0.1 s. Rolling collision energy voltage was used. The top 20 parent ions were selected following MS/MS. Mascot (version 2.3.02) was used to search the database.

#### Proteomics identifications (PRIDE)

The released proteins were identified by the methods described above. Under LC conditions, the gradient was as follows: 0–4.5 min 95% A, 4.5–95 min 95–70% A, 95–101 min 70–40% A, 101–106 min 40–10% A, 106–131 min 10% A, 131–132 min 10–95% A, and 132–150 min 95% A. The flow rate was 300 nL/min at 35 °C, and the sample loading volume was 10 μL (0.1% FA).

The mass spectrometric conditions were as follows: The operating parameters of ESI mode were utilized: ion spray voltage + 2800 V, interface heater temperature 150 °C, declustering potential 80 V, column oven temperature 35 °C. The sheath gas and curtain gas were both nitrogen, and the pressures were 15 and 30 psi, respectively. The IDA mode was utilized. The TOF–MS scan range was set to 350–1250 m/z acclimated for 0.25 s, and the MS/MS scan range was set to 65–1800 m/z acclimated for 0.1 s. Rolling collision energy voltage was used. The top 20 parent ions were selected following MS/MS. Mascot (version 2.3.02) was used to search the database.

#### Mascot search parameters

The type of search and enzyme were set as MS/MS Ion Search and trypsin, respectively. The variable modifications included carbamidomethyl (C), deamidated (NQ), oxidation (HW), and oxidation (M). Monoisotopic was used as the mass parameter, and the mass tolerance values for peptides and fragments were ± 0.05 Da and ± 0.03 Da, respectively. The max missed cleavages by enzyme was set as 2. The database used for the search was the NCBI *K. marxianus* database. The results are provided as a Supplementary Dataset.

### Quantitative real time polymerase chain reaction procedure

Total RNA extraction was performed using TRIzol Reagent (Invitrogen). The yeast cells were collected by centrifugation, and then, the total RNA was extracted according to the manufacturer’s instructions. The RNA yield was determined by a Nano-100 Micro-Spectrophotometer (Medclub Scientific Co., Ltd., Taiwan). A total of 0.1 μg of RNA was converted to cDNA using the GoScript™ Reverse Transcription System, and qPCR analysis was performed using LightCycler 480 SYBR Green Master Mix (Roche) on a Rotor-Gene 3000 Real-Time DNA Detection System (Corbett Research). The transcription levels were calculated for reverse transcriptase-treated and nontreated samples and normalized to those of actin (*ACT*). The changes in relative transcription levels were calculated as fold changes using the ΔΔCt method. All the primers used for analysis in this study are listed in Supplementary Table S5.

## Supplementary Information

Supplementary Information 1.

Supplementary Information 2.

## Data Availability

All data generated or analyzed during this study are included in this published article (and its Supplementary Information files).
