# Supplementary material for: Growth and autolysis of the kefir yeast Kluyveromyces marxianus in lactate culture
Source: Sci Rep. 2021 Jul 15;11:14552. doi: 10.1038/s41598-021-94101-y (PMC8282799; doi:10.1038/s41598-021-94101-y)

Growth and autolysis of the kefir yeast *Kluyveromyces marxianus* in lactate culture

Shou-Chen Lo[1*], Chia-Yin Yang[1], Dony Chacko Mathew[2], and Chieh-Chen Huang[1,3,4*]

[1]Department of Life Sciences, National Chung Hsing University, Taichung, 402, Taiwan

[2] Washington High School, Taichung, 406, Taiwan

[3] Program in Microbial Genomics, National Chung Hsing University, Taichung, 402, Taiwan

[4]Innovation and Development Center of Sustainable Agriculture, National Chung Hsing University, Taichung, 402, Taiwan

*Cocorresponding authors: Chieh-Chen Huang, Ph.D., and Shou-Chen Lo, Ph.D.

E-mail addresses: cchuang@dragon.nchu.edu.tw (C.C.H.) and scl@dragon.nchu.edu.tw (S.C.L.)

Mailing address: Life Sciences Building 4F. R.403, No.145, Xingda Rd., Taichung

402, Taiwan

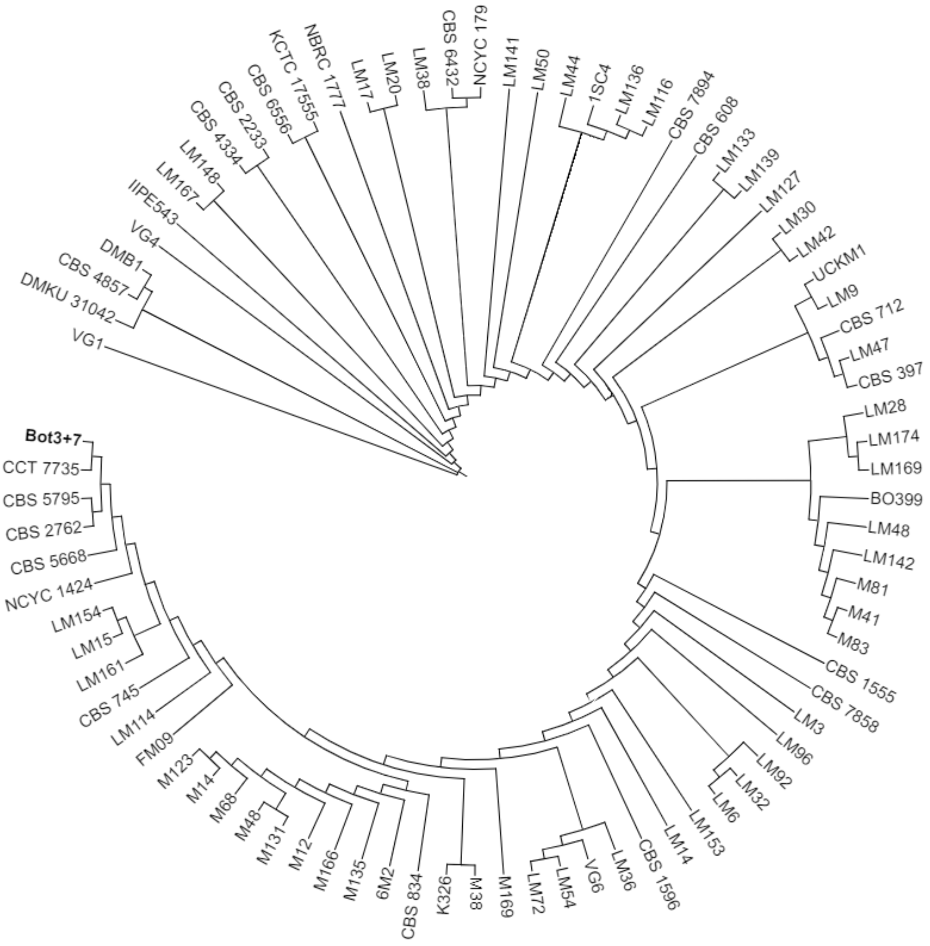

Figure S1. The phylogenetic tree of *K. marxianus* Bot3+7 and other *K. marxianus* strains. The phylogenetic tree was generated with Interactive Tree Of Life online tool (version 6.0) under free access mode (itol.embl.de).

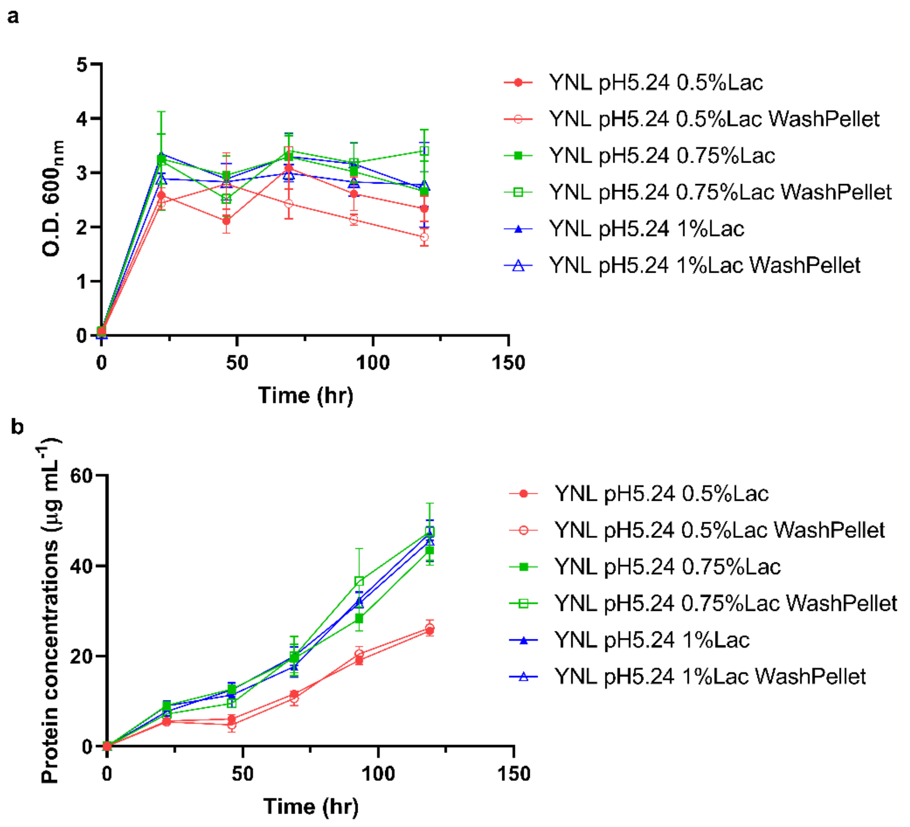

Figure S2. Growth curves and released protein concentrations of *K. marxianus* strain Bot3+7 at low lactic acid concentrations. (a) Growth curve of *K. marxianus* strain Bot3+7 in the presence of 0.5%, 0.75% and 1% lactic acid in YNL medium. (b) Released protein concentration of K. marxianus strain Bot3+7 in the presence of 0.5%, 0.75% and 1% lactic acid in YNL medium. The data are presented as the mean and standard deviation of three replicates. This image was created by using GraphPad Prism version 8.2.1 (https://www.graphpad.com/scientific-software/prism/).

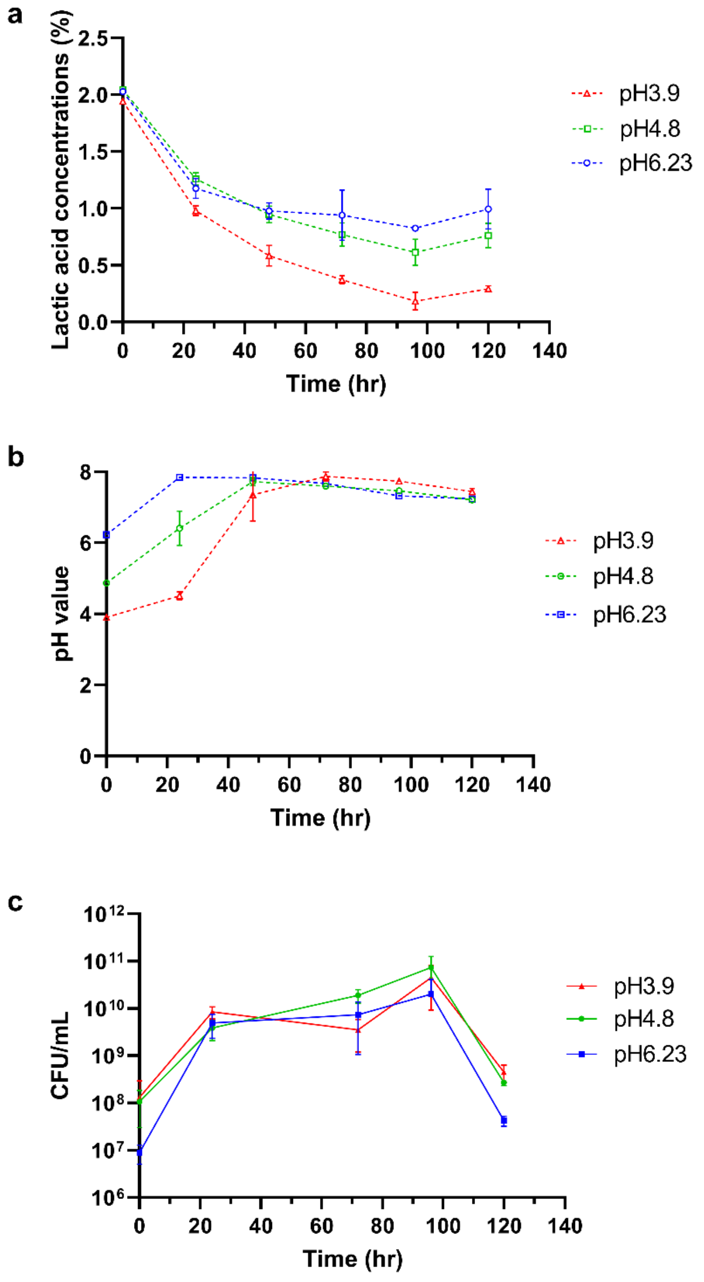

Figure S3. The lactic acid concentration (a), pH values (b) and CFU/mL (c) changes of the *K. marxianus* Bot3+7 strain in YNL culture at initial pH 3.9, pH 4.8 and pH 6.23. The data are presented as the mean and standard deviation of three replicates. This image was created by using GraphPad Prism (version 8.2.1; https://www.graphpad.com/scientific-software/prism/).

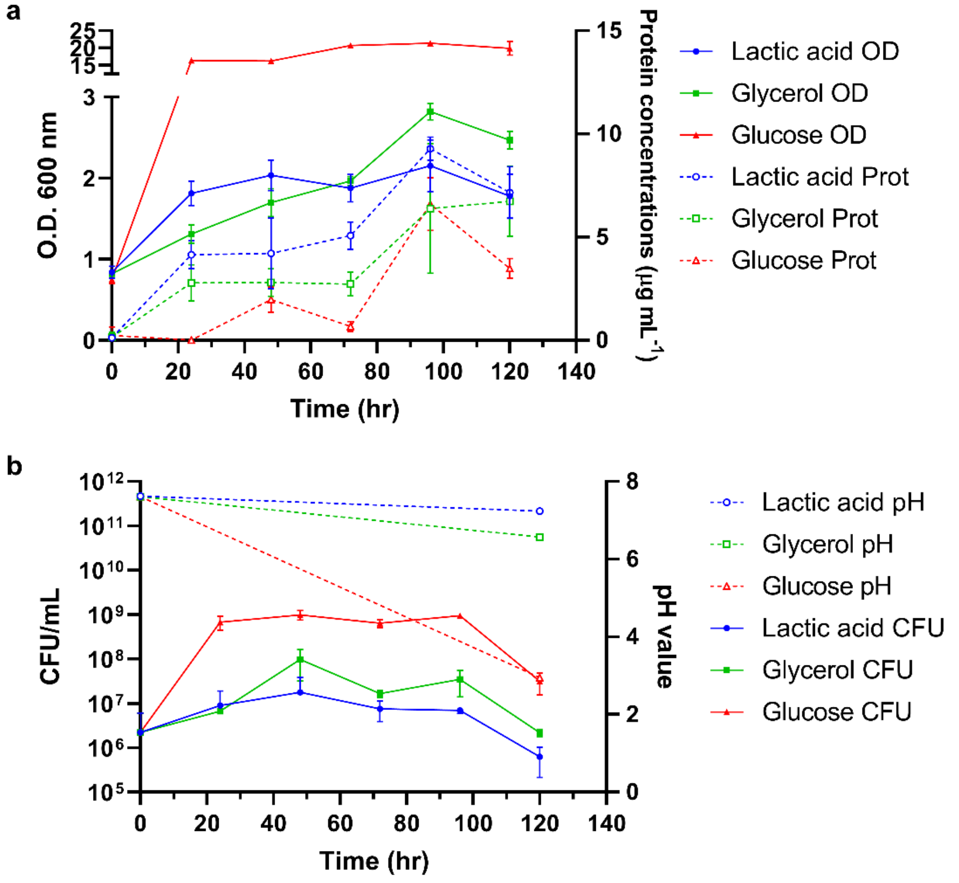

Figure S4. (a) The growth and released protein concentrations of the *K. marxianus* Bot3+7 strain with different carbon sources at initial pH 7.6 culture. (b) The CFU and pH values changes of the *K. marxianus* Bot3+7 strain with different carbon sources at initial pH 7.6 culture. The data are presented as the mean and standard deviation of three replicates. This image was created by using GraphPad Prism (version 8.2.1; https://www.graphpad.com/scientific-software/prism/).

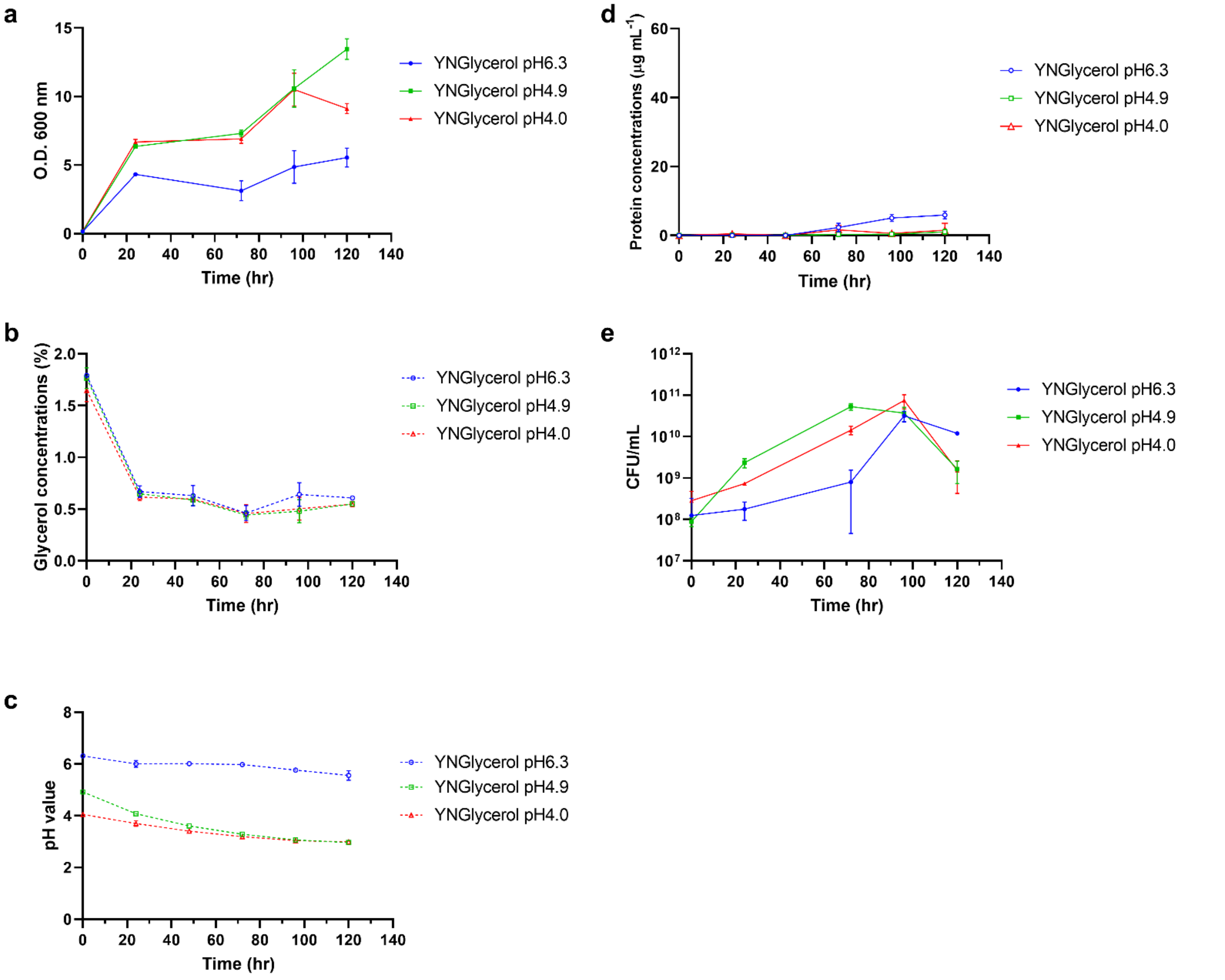

Figure S5. The growth (a), glycerol concentrations (b), pH values (c), protein concentrations (d) and colony-forming unit (e) of *K. marxianus* Bot3+7 strain culture with glycerol at initial pH 4.0, pH 4.9 and pH 6.3. The data are presented as the mean and standard deviation of three replicates. This image was created by using GraphPad Prism (version 8.2.1; https://www.graphpad.com/scientific-software/prism/).

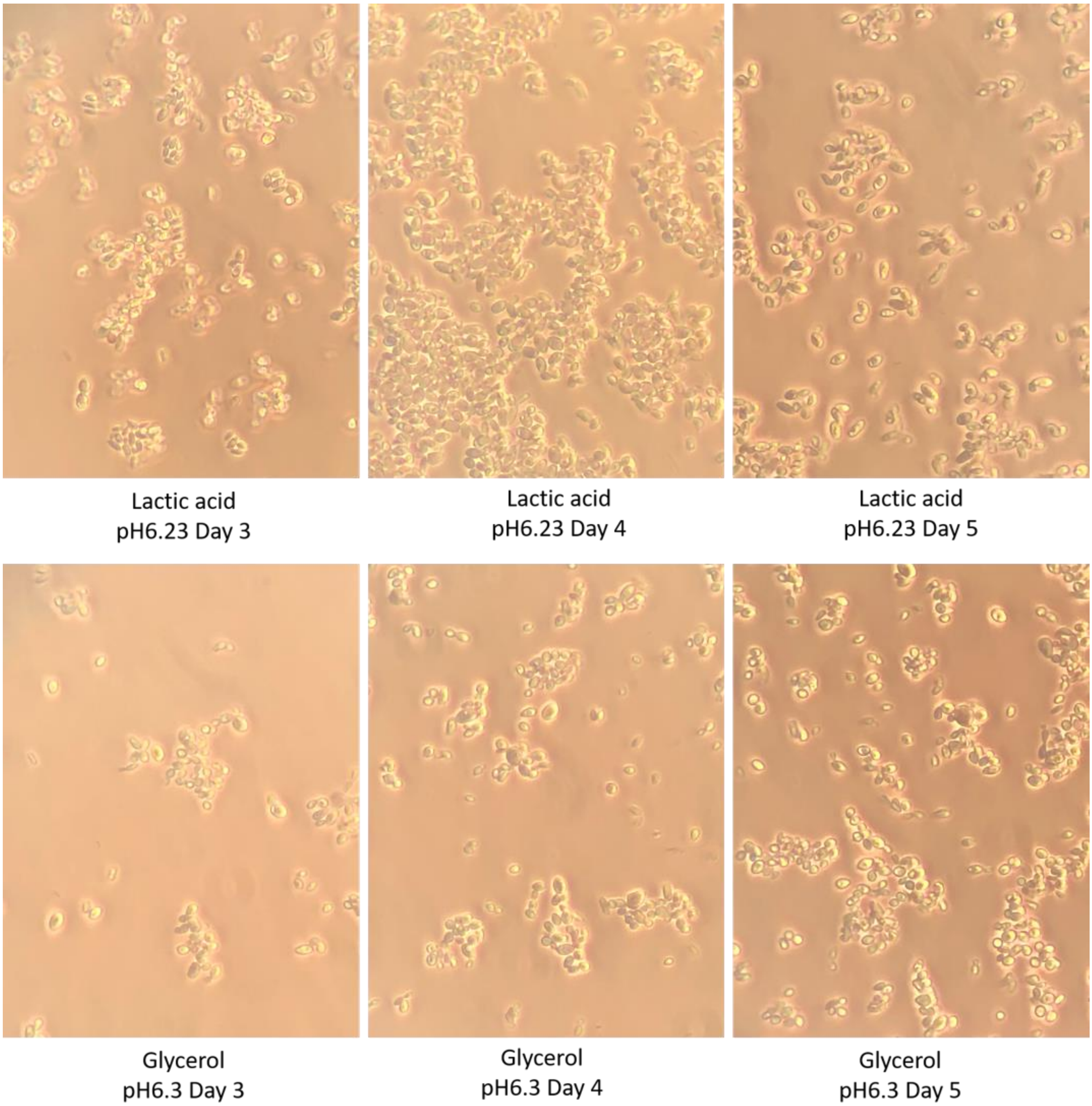

Figure S6. The microscope photographs of *K. marxianus* Bot3+7 strain from lactic acid and glycerol cultures at initial pH 6.23 and pH 6.3, respectively. The photographs are uncropped. The image was taken by an iPhone 8 (Camera app in iOS 14.6, Apple).

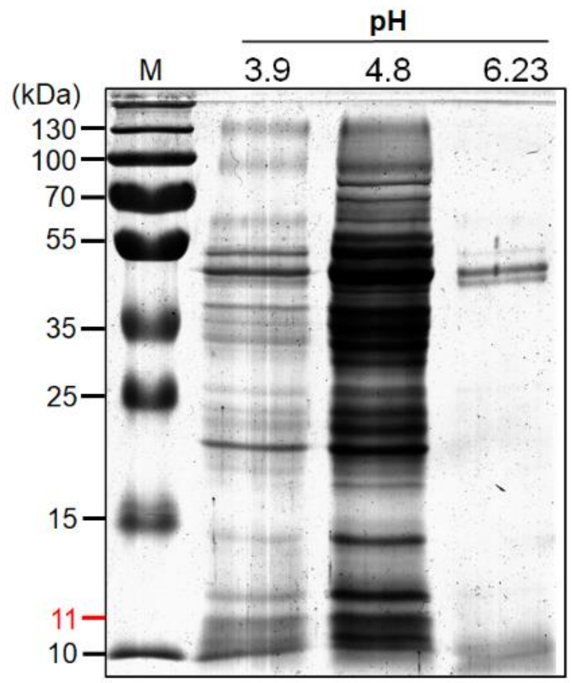

Figure S7. The SDS-PAGE analysis of 20 folds concentrated proteins from YNL culture filtrate at pH 3.9, pH 4.8 and pH 6.23. This image was edited by using Microsoft Office Professional 2019 PowerPoint (https://www.microsoft.com/zh-tw/microsoft-365/p/office-%E5%B0%88%E6%A5%AD%E7%89%88-2019/cfq7ttc0k7c5?activetab=pivot%3aoverviewtab).

Supplementary Table S1. LC-MS/MS analysis of the 37 kDa and 27 kDa proteins from SDS-PAGE.

Molecular weight[a]	Protein name[b]	Matched sequences[c]
37 kDa	Glyceraldehyde 3-phosphate dehydrogenase 1 in *Kluyveromyces marxianus* (P84998)	**1** MVSIAINGFG RIGRLVLRIA LERKNIDVVA INDPFISVDY AAYMFKYDST<br>**51** HGKYKGEVSH DGSNLIINGK K**VAVFQEK**DP ATLPWGKLGV DIAVDSTGVF<br>**101** KELDSAQKHI DAGAKK**VVIT APSK**TAPMFV VGVNEDKYNG EKIVSNASCT<br>**151** TNCLAPIAKI INDEFGIEEG LMTTVHSITA TQKTVDGPSH KDWRGGR**TAS**<br>**201 GNIIPSSTGA AK**AVGK**VLPE LQGKLTGMAF R**VPTTDVSVV DLTVKLVK**AA**<br>**251 TYDEIK**AAVK KVSEGKLKDV VGYTEDAVVS SDFLGDTHST IFDAAAGIQL<br>**301** SPKFVKLVAW YDNEYGYSTR VVDLVEHVA
27 kDa	Glyceraldehyde 3-phosphate dehydrogenase 1 in *Kluyveromyces marxianus* (P84998)	**1** MVSIAINGFG RIGRLVLRIA LERKNIDVVA INDPFISVDY AAYMFKYDST<br>**51** HGKYKGEVSH DGSNLIINGK KVAVFQEKDP ATLPWGKLGV DIAVDSTGVF<br>**101** KELDSAQKHI DAGAKK**VVIT APSK**TAPMFV VGVNEDKYNG EKIVSNASCT<br>**151** TNCLAPIAKI INDEFGIEEG LMTTVHSITA TQKTVDGPSH KDWRGGR**TAS**<br>**201 GNIIPSSTGA AK**AVGKVLPE LQGKLTGMAF RVPTTDVSVV DLTVKLVK**AA**<br>**251 TYDEIK**AAVK KVSEGKLKDV VGYTEDAVVS SDFLGDTHST IFDAAAGIQL<br>**301** SPKFVKLVAW YDNEYGYSTR VVDLVEHVA

[a]The protein bands that were excised from the SDS-PAGE gel.

[b]The names and accession numbers of the matched proteins in NBCI are presented.

[c]The matched peptides are shown in bold, and the acid cleavage sites (aspartic acid-proline, D-P) are underlined.

Supplementary Table S2. The identified proteins from 120 hours lactate cultured *Kluyveromyces marxianus* cell pellet by LC-MS/MS analysis.

Metabolism	UniProt accession number	Protein name	Gene Ontology (Biological process)
Tricarboxylic acid cycle	W0TDY2	2-oxoglutarate dehydrogenase E1 component	tricarboxylic acid cycle [GO:0006099]
	W0T7K6	Aconitate hydratase, mitochondrial (Aconitase) (EC 4.2.1.-)	mitochondrial genome maintenance [GO:0000002]; tricarboxylic acid cycle [GO:0006099]
	W0THE4	Malate synthase (EC 2.3.3.9)	glyoxylate cycle [GO:0006097]; tricarboxylic acid cycle [GO:0006099]
	W0TFA8	Citrate synthase	propionate catabolic process, 2-methylcitrate cycle [GO:0019629]; tricarboxylic acid cycle [GO:0006099]
	W0TEG4	Isocitrate dehydrogenase [NADP] (EC 1.1.1.42)	glutamate biosynthetic process [GO:0006537]; isocitrate metabolic process [GO:0006102]; tricarboxylic acid cycle [GO:0006099]
	W0TJM6	Fumarate hydratase (EC 4.2.1.2)	double-strand break repair [GO:0006302]; fumarate metabolic process [GO:0006106]; tricarboxylic acid cycle [GO:0006099]
	W0TEG9	Malate dehydrogenase (EC 1.1.1.37)	carbohydrate metabolic process [GO:0005975]; malate metabolic process [GO:0006108]; tricarboxylic acid cycle [GO:0006099]
	W0TBX1	Dihydrolipoyllysine-residue succinyltransferase (EC 2.3.1.61)	L-lysine catabolic process to acetyl-CoA via saccharopine [GO:0033512]; tricarboxylic acid cycle [GO:0006099]
	W0TGG7	Isocitrate dehydrogenase [NAD] subunit, mitochondrial	isocitrate metabolic process [GO:0006102]; tricarboxylic acid cycle [GO:0006099]
	W0TA03	Succinate dehydrogenase [ubiquinone] flavoprotein subunit, mitochondrial (EC 1.3.5.1)	electron transport chain [GO:0022900]; tricarboxylic acid cycle [GO:0006099]
	W0T4J0	Succinate--CoA ligase [ADP-forming] subunit beta, mitochondrial (EC 6.2.1.5) (Succinyl-CoA synthetase beta chain) (SCS-beta)	succinyl-CoA metabolic process [GO:0006104]; tricarboxylic acid cycle [GO:0006099]
	W0T883	Succinate--CoA ligase [ADP-forming] subunit alpha, mitochondrial (EC 6.2.1.5) (Succinyl-CoA synthetase subunit alpha) (SCS-alpha)	succinyl-CoA metabolic process [GO:0006104]; tricarboxylic acid cycle [GO:0006099]
	W0T7K3	Isocitrate dehydrogenase [NAD] subunit, mitochondrial	tricarboxylic acid cycle [GO:0006099]
	W0TD84	Malate dehydrogenase (EC 1.1.1.37)	carbohydrate metabolic process [GO:0005975]; fatty acid beta-oxidation [GO:0006635]; malate metabolic process [GO:0006108]; NADH regeneration [GO:0006735]; tricarboxylic acid cycle [GO:0006099]
	W0T2Z0	Aconitate hydratase, mitochondrial (Aconitase) (EC 4.2.1.-)	tricarboxylic acid cycle [GO:0006099]
	W0TKL8	Succinate dehydrogenase [ubiquinone] iron-sulfur subunit, mitochondrial (EC 1.3.5.1)	tricarboxylic acid cycle [GO:0006099]
	W0TAI6	Malate dehydrogenase (EC 1.1.1.37)	carboxylic acid metabolic process [GO:0019752]; gluconeogenesis [GO:0006094]; protein import into peroxisome matrix [GO:0016558]; tricarboxylic acid cycle [GO:0006099]
Glycolysis/gluconeogenesis	W0TD31	Phosphoenolpyruvate carboxykinase (ATP) (EC 4.1.1.49)	gluconeogenesis [GO:0006094]
	W0T3B0	Pyruvate carboxylase (EC 6.4.1.1)	gluconeogenesis [GO:0006094]; pyruvate metabolic process [GO:0006090]
	W0T3H5	Phosphoglycerate kinase (EC 2.7.2.3)	gluconeogenesis [GO:0006094]; glycolytic process [GO:0006096]
	W0TDE9	Triosephosphate isomerase (EC 5.3.1.1)	gluconeogenesis [GO:0006094]; glycolytic process [GO:0006096]
	Q70JN8	Triosephosphate isomerase (TIM) (EC 5.3.1.1)	gluconeogenesis [GO:0006094]; glycolytic process [GO:0006096]

		(Triose-phosphate isomerase)	
	W0TF96	Fructose-bisphosphatase (EC 3.1.3.11)	cellular response to glucose starvation [GO:0042149]; gluconeogenesis [GO:0006094]; reactive oxygen species metabolic process [GO:0072593]
	W0T9W3	Fructose-bisphosphate aldolase (FBP aldolase) (EC 4.1.2.13)	gluconeogenesis [GO:0006094]; glycolytic process [GO:0006096]
	W0T4R5	Glucose-6-phosphate isomerase (EC 5.3.1.9)	gluconeogenesis [GO:0006094]; glycolytic process [GO:0006096]
	W0TAR9	Ubiquitin carboxyl-terminal hydrolase (EC 3.4.19.12)	negative regulation of gluconeogenesis [GO:0045721]; proteasome-mediated ubiquitin-dependent protein catabolic process [GO:0043161]; protein deubiquitination [GO:0016579]
	W0TAI6	Malate dehydrogenase (EC 1.1.1.37)	carboxylic acid metabolic process [GO:0019752]; gluconeogenesis [GO:0006094]; protein import into peroxisome matrix [GO:0016558]; tricarboxylic acid cycle [GO:0006099]
	P84998	Glyceraldehyde-3-phosphate dehydrogenase 1 (GAPDH 1) (EC 1.2.1.12)	glucose metabolic process [GO:0006006]; glycolytic process [GO:0006096]
	A4ZGQ9	Glyceraldehyde-3-phosphate dehydrogenase (EC 1.2.1.12)	glucose metabolic process [GO:0006006]; glycolytic process [GO:0006096]
	W0T7K9	Phosphopyruvate hydratase (EC 4.2.1.11)	glycolytic process [GO:0006096]
	W0TDT2	Pyruvate kinase (EC 2.7.1.40)	canonical glycolysis [GO:0061621]; pyruvate biosynthetic process [GO:0042866]
	W0TF55	Phosphotransferase (EC 2.7.1.-)	cellular glucose homeostasis [GO:0001678]; glycolytic process [GO:0006096]
	W0T3P9	Phosphotransferase (EC 2.7.1.-)	cellular glucose homeostasis [GO:0001678]; fructose 6-phosphate metabolic process [GO:0006002]; glycolytic fermentation [GO:0019660]; glycolytic process [GO:0006096]; mannose metabolic process [GO:0006013]
Pentose-phosphate pathway	W0TCV6	Transaldolase (EC 2.2.1.2)	carbohydrate metabolic process [GO:0005975]; pentose-phosphate shunt [GO:0006098]
	W0T3V9	6-phosphogluconate dehydrogenase, decarboxylating (EC 1.1.1.44)	D-gluconate metabolic process [GO:0019521]; pentose-phosphate shunt [GO:0006098]
	W0T4I6	6-phosphogluconolactonase-like protein	carbohydrate metabolic process [GO:0005975]; pentose-phosphate shunt [GO:0006098]
Galactose metabolism	S5WA50	UDP-glucose 4-epimerase	galactose metabolic process [GO:0006012]
Other carbohydrate metabolism	W0TBD3	Phosphoglucomutase-2	carbohydrate metabolic process [GO:0005975]
	B3GQU5	D-arabinose dehydrogenase [NAD(P)+] heavy chain	cellular carbohydrate metabolic process [GO:0044262]
	W0T4T5	Glucose-6-phosphate 1-epimerase (EC 5.1.3.15)	carbohydrate metabolic process [GO:0005975]
Lactate metabolism	W0T5A6	Cytochrome b2	lactate metabolic process [GO:0006089]
	W0TC20	D-lactate dehydrogenase [cytochrome]	lactate catabolic process [GO:1903457]
	W0TFC1	D-lactate dehydrogenase [cytochrome] 2	lactate catabolic process [GO:1903457]
	W0TGY6	Carboxylic acid transporter protein homolog	plasma membrane lactate transport [GO:0035879]; plasma membrane pyruvate transport [GO:0006849]; plasma membrane selenite transport [GO:0097080]
**Stress response**			
Starvation	W0TF96	Fructose-bisphosphatase (EC 3.1.3.11)	cellular response to glucose starvation [GO:0042149]; gluconeogenesis [GO:0006094]; reactive oxygen species metabolic process [GO:0072593]
	W0TBM6	Sodium transport ATPase 5	cellular response to glucose starvation [GO:0042149]; hyperosmotic response [GO:0006972]; response to pH [GO:0009268]; response to salt stress [GO:0009651]
	W0T855	cAMP-dependent protein kinase regulatory subunit	cAMP-mediated signaling [GO:0019933]; negative regulation of meiotic cell cycle [GO:0051447]; negative regulation of Ras protein signal transduction [GO:0046580]; positive regulation of adenylate cyclase activity [GO:0045762]; positive regulation of protein export from nucleus [GO:0046827]; positive regulation of transcription from RNA polymerase II promoter in response to

			glucose starvation [GO:0061406]; positive regulation of transcription from RNA polymerase II promoter in response to nitrogen starvation [GO:0036278]; protein localization to bud neck [GO:0097271]; regulation of cytoplasmic mRNA processing body assembly [GO:0010603]
Osmotic	W0TAV4	12 kDa heat shock protein	cell adhesion [GO:0007155]; cellular response to heat [GO:0034605]; cellular response to osmotic stress [GO:0071470]; cellular response to oxidative stress [GO:0034599]; plasma membrane organization [GO:0007009]
	W0TBM6	Sodium transport ATPase 5	cellular response to glucose starvation [GO:0042149]; hyperosmotic response [GO:0006972]; response to pH [GO:0009268]; response to salt stress [GO:0009651]
	W0T9K6	Nuclear cap-binding protein complex subunit 1	mRNA cis splicing, via spliceosome [GO:0045292]; mRNA transport [GO:0051028]; nuclear-transcribed mRNA catabolic process, nonsense-mediated decay [GO:0000184]; response to osmotic stress [GO:0006970]
Oxidative stress	W0TAV4	12 kDa heat shock protein	cell adhesion [GO:0007155]; cellular response to heat [GO:0034605]; cellular response to osmotic stress [GO:0071470]; cellular response to oxidative stress [GO:0034599]; plasma membrane organization [GO:0007009]
	W0TIE1	Actin	ascospore wall assembly [GO:0030476]; cellular response to oxidative stress [GO:0034599]; DNA repair [GO:0006281]; endocytosis [GO:0006897]; establishment of cell polarity [GO:0030010]; mitotic actomyosin contractile ring contraction [GO:1902404]; protein secretion [GO:0009306]; vacuole inheritance [GO:0000011]
	W0TC44	Peroxiredoxin TSA1	cell redox homeostasis [GO:0045454]; cellular detoxification of hydrogen peroxide [GO:0061692]; hydrogen peroxide catabolic process [GO:0042744]; negative regulation of DNA-binding transcription factor activity [GO:0043433]; positive regulation of transcription from RNA polymerase II promoter in response to oxidative stress [GO:0036091]
	W0T804	NADH-cytochrome b5 reductase (EC 1.6.2.2)	cellular response to oxidative stress [GO:0034599]; ergosterol biosynthetic process [GO:0006696]
	W0TKG9	Mitochondrial peroxiredoxin PRX1	cell redox homeostasis [GO:0045454]; cellular response to oxidative stress [GO:0034599]; regulation of hydrogen peroxide-induced cell death [GO:1903205]; response to cadmium ion [GO:0046686]
	W0T8K0	Glutathione reductase (EC 1.8.1.7)	cell redox homeostasis [GO:0045454]; cellular response to menadione [GO:0036245]; cellular response to oxidative stress [GO:0034599]; glutathione metabolic process [GO:0006749]; protein glutathionylation [GO:0010731]
	W0TDP7	NADPH-dependent alpha-keto amide reductase	cellular amide metabolic process [GO:0043603]; cellular aromatic compound metabolic process [GO:0006725]; cellular ketone metabolic process [GO:0042180]; cellular response to oxidative stress [GO:0034599]
	W0TG01	Translationally-controlled tumor protein homolog	cellular response to oxidative stress [GO:0034599]; cytoplasmic translation [GO:0002181]; negative regulation of autophagy [GO:0010507]; negative regulation of microtubule depolymerization [GO:0007026]
	W0TFP1	Peroxidase (EC 1.11.1.-)	cellular response to oxidative stress [GO:0034599]
	W0TF38	Succinate-semialdehyde dehydrogenase (EC 1.2.1.16)	cellular response to oxidative stress [GO:0034599]; gamma-aminobutyric acid catabolic process [GO:0009450]; glutamate decarboxylation to succinate [GO:0006540]
	W0TAN7	Glutamate decarboxylase (EC 4.1.1.15)	cellular response to oxidative stress [GO:0034599]; glutamate catabolic process [GO:0006538]
Protein folding	W0T5P3	Heat shock protein 60	'de novo' protein folding [GO:0006458]; chaperone-mediated protein complex assembly [GO:0051131]; protein import into mitochondrial intermembrane space [GO:0045041]; protein maturation [GO:0051604]; protein refolding [GO:0042026]; protein stabilization [GO:0050821]
	W0THY0	Heat shock protein SSA3	protein folding [GO:0006457]; SRP-dependent cotranslational protein targeting to membrane, translocation [GO:0006616]
	W0T8D4	Heat shock protein SSC1	protein folding [GO:0006457]
	W0TAQ8	Heat shock protein 104	cellular heat acclimation [GO:0070370]; chaperone cofactor-dependent protein refolding [GO:0051085]; protein folding in endoplasmic reticulum [GO:0034975]; protein unfolding [GO:0043335]; stress granule disassembly [GO:0035617]; trehalose metabolism in response to heat stress [GO:0070414]
	W0T4B9	Protein disulfide-isomerase (EC 5.3.4.1)	mannose trimming involved in glycoprotein ERAD pathway [GO:1904382]; protein folding [GO:0006457]
	W0TCK6	Peptidyl-prolyl cis-trans isomerase (PPIase) (EC	ascospore formation [GO:0030437]; histone deacetylation [GO:0016575]; positive regulation of meiotic nuclear division

		5.2.1.8)	[GO:0045836]; protein folding [GO:0006457]
	W0TIR2	Putative aspartyl aminopeptidase	chaperone-mediated protein folding [GO:0061077]
	W0TBW3	Prohibitin	inner mitochondrial membrane organization [GO:0007007]; mitochondrion inheritance [GO:0000001]; mitochondrion morphogenesis [GO:0070584]; negative regulation of proteolysis [GO:0045861]; protein folding [GO:0006457]
	W0TD70	ATP-dependent molecular chaperone HSC82	adenylate cyclase-activating glucose-activated G protein-coupled receptor signaling pathway [GO:0010619]; chaperone-mediated protein folding [GO:0061077]; heterochromatin assembly by small RNA [GO:0031048]
	W0TBY6	Peptidyl-prolyl cis-trans isomerase (PPIase) (EC 5.2.1.8)	apoptotic process [GO:0006915]; protein folding [GO:0006457]
	W0T5P8	Protein SIS1	misfolded protein transport [GO:0070843]; mitochondria-associated ubiquitin-dependent protein catabolic process [GO:0072671]; nuclear protein quality control by the ubiquitin-proteasome system [GO:0071630]; protein folding [GO:0006457]; translational initiation [GO:0006413]; tRNA import into nucleus [GO:0035719]
	W0TE64	Prohibitin	mitochondrion inheritance [GO:0000001]; negative regulation of proteolysis [GO:0045861]; protein folding [GO:0006457]
	W0T9M6	T-complex protein 1 subunit gamma	protein folding [GO:0006457]
	W0T847	DnaJ homolog 1	'de novo' protein folding [GO:0006458]; mitochondrial genome maintenance [GO:0000002]; protein quality control for misfolded or incompletely synthesized proteins [GO:0006515]; protein refolding [GO:0042026]; response to heat [GO:0009408]
	W0TC41	CCT-beta	protein folding [GO:0006457]

Supplementary Table S3. The identified proteins from 120 hours lactate cultured *Kluyveromyces marxianus* medium by LC-MS/MS analysis.

Metabolism	UniProt accession number	Protein name	Gene Ontology (Biological process)
Tricarboxylic acid cycle	W0TEG9	Malate dehydrogenase (EC 1.1.1.37)	carbohydrate metabolic process [GO:0005975]; malate metabolic process [GO:0006108]; tricarboxylic acid cycle [GO:0006099]
	W0T7K6	Aconitate hydratase, mitochondrial (Aconitase) (EC 4.2.1.-)	mitochondrial genome maintenance [GO:0000002]; tricarboxylic acid cycle [GO:0006099]
	W0THE4	Malate synthase (EC 2.3.3.9)	glyoxylate cycle [GO:0006097]; tricarboxylic acid cycle [GO:0006099]
	W0T883	Succinate--CoA ligase [ADP-forming] subunit alpha, mitochondrial (EC 6.2.1.5) (Succinyl-CoA synthetase subunit alpha) (SCS-alpha)	succinyl-CoA metabolic process [GO:0006104]; tricarboxylic acid cycle [GO:0006099]
	W0TFA8	Citrate synthase	propionate catabolic process, 2-methylcitrate cycle [GO:0019629]; tricarboxylic acid cycle [GO:0006099]
	W0T7K3	Isocitrate dehydrogenase [NAD] subunit, mitochondrial	tricarboxylic acid cycle [GO:0006099]
	W0TAI6	Malate dehydrogenase (EC 1.1.1.37)	carboxylic acid metabolic process [GO:0019752]; gluconeogenesis [GO:0006094]; protein import into peroxisome matrix [GO:0016558]; tricarboxylic acid cycle [GO:0006099]
	W0TA03	Succinate dehydrogenase [ubiquinone] flavoprotein subunit, mitochondrial (EC 1.3.5.1)	electron transport chain [GO:0022900]; tricarboxylic acid cycle [GO:0006099]
	W0TGG7	Isocitrate dehydrogenase [NAD] subunit, mitochondrial	isocitrate metabolic process [GO:0006102]; tricarboxylic acid cycle [GO:0006099]
	W0T4J0	Succinate--CoA ligase [ADP-forming] subunit beta, mitochondrial (EC 6.2.1.5) (Succinyl-CoA synthetase beta chain) (SCS-beta)	succinyl-CoA metabolic process [GO:0006104]; tricarboxylic acid cycle [GO:0006099]
	W0T2Z0	Aconitate hydratase, mitochondrial (Aconitase) (EC 4.2.1.-)	tricarboxylic acid cycle [GO:0006099]
	W0TDY2	2-oxoglutarate dehydrogenase E1 component	tricarboxylic acid cycle [GO:0006099]
Glycolysis/gluconeogenesis	W0T3H5	Phosphoglycerate kinase (EC 2.7.2.3)	gluconeogenesis [GO:0006094]; glycolytic process [GO:0006096]
	W0T9W3	Fructose-bisphosphate aldolase (FBP aldolase) (EC 4.1.2.13)	gluconeogenesis [GO:0006094]; glycolytic process [GO:0006096]
	W0T4R5	Glucose-6-phosphate isomerase (EC 5.3.1.9)	gluconeogenesis [GO:0006094]; glycolytic process [GO:0006096]
	W0TF96	Fructose-bisphosphatase (EC 3.1.3.11)	cellular response to glucose starvation [GO:0042149]; gluconeogenesis [GO:0006094]; reactive oxygen species metabolic process [GO:0072593]
	W0TAI6	Malate dehydrogenase (EC 1.1.1.37)	carboxylic acid metabolic process [GO:0019752]; gluconeogenesis [GO:0006094]; protein import into peroxisome matrix [GO:0016558]; tricarboxylic acid cycle [GO:0006099]
	W0T7K9	Phosphopyruvate hydratase (EC 4.2.1.11)	glycolytic process [GO:0006096]
	W0T9W3	Fructose-bisphosphate aldolase (FBP aldolase) (EC 4.1.2.13)	gluconeogenesis [GO:0006094]; glycolytic process [GO:0006096]
	P84998	Glyceraldehyde-3-phosphate dehydrogenase 1 (GAPDH 1) (EC 1.2.1.12)	glucose metabolic process [GO:0006006]; glycolytic process [GO:0006096]
	W0T4R5	Glucose-6-phosphate isomerase (EC 5.3.1.9)	gluconeogenesis [GO:0006094]; glycolytic process [GO:0006096]
	W0TF55	Phosphotransferase (EC 2.7.1.-)	cellular glucose homeostasis [GO:0001678]; glycolytic process [GO:0006096]
	W0T3P9	Phosphotransferase (EC	cellular glucose homeostasis [GO:0001678]; fructose 6-phosphate

		2.7.1.-)	metabolic process [GO:0006002]; glycolytic fermentation [GO:0019660]; glycolytic process [GO:0006096]; mannose metabolic process [GO:0006013]
	W0T6W0	Phosphoglycerate mutase 3	glycolytic process [GO:0006096]
Pentose-phosphate pathway	W0TCV6	Transaldolase (EC 2.2.1.2)	carbohydrate metabolic process [GO:0005975]; pentose-phosphate shunt [GO:0006098]
	W0TCY6	Glucose-6-phosphate 1-dehydrogenase (EC 1.1.1.49)	glucose metabolic process [GO:0006006]; pentose-phosphate shunt [GO:0006098]
	W0T3V9	6-phosphogluconate dehydrogenase, decarboxylating (EC 1.1.1.44)	D-gluconate metabolic process [GO:0019521]; pentose-phosphate shunt [GO:0006098]
Galactose metabolism	W0T4K1	NAD(P)H-dependent D-xylose reductase	arabinose catabolic process [GO:0019568]; cellular response to osmotic stress [GO:0071470]; cellular response to oxidative stress [GO:0034599]; D-xylose catabolic process [GO:0042843]; galactose catabolic process [GO:0019388]
Other carbohydrate metabolism	W0TBD3	Phosphoglucomutase-2	carbohydrate metabolic process [GO:0005975]
	W0TK22	Probable family 17 glucosidase SCW4	carbohydrate metabolic process [GO:0005975]
	B3GQU5	Exo-inulinase (EC 3.2.1.7) (Inulinase)	carbohydrate metabolic process [GO:0005975]
	W0T4T5	Glycerol-3-phosphate dehydrogenase [NAD(+)] (EC 1.1.1.8)	carbohydrate metabolic process [GO:0005975]; glycerol-3-phosphate catabolic process [GO:0046168]
	W0T8C0	Glucose-6-phosphate 1-epimerase (EC 5.1.3.15)	carbohydrate metabolic process [GO:0005975]
	W0TC08	Glycosidase (EC 3.2.-.-)	carbohydrate metabolic process [GO:0005975]; cell wall chitin metabolic process [GO:0006037]; fungal-type cell wall organization [GO:0031505]
Lactate metabolism	W0TFC1	D-lactate dehydrogenase [cytochrome] 2	lactate catabolic process [GO:1903457]
	W0T5A6	Cytochrome b2	lactate metabolic process [GO:0006089]
**Stress response**			
Starvation	W0TF96	Fructose-bisphosphatase (EC 3.1.3.11)	cellular response to glucose starvation [GO:0042149]; gluconeogenesis [GO:0006094]; reactive oxygen species metabolic process [GO:0072593]
	W0T7F7	Cystathionine beta-synthase (EC 4.2.1.22)	cysteine biosynthetic process from serine [GO:0006535]; cysteine biosynthetic process via cystathionine [GO:0019343]; hydrogen sulfide biosynthetic process [GO:0070814]; transsulfuration [GO:0019346]; traversing start control point of mitotic cell cycle [GO:0007089]
	W0T855	cAMP-dependent protein kinase regulatory subunit	cAMP-mediated signaling [GO:0019933]; negative regulation of meiotic cell cycle [GO:0051447]; negative regulation of Ras protein signal transduction [GO:0046580]; positive regulation of adenylate cyclase activity [GO:0045762]; positive regulation of protein export from nucleus [GO:0046827]; positive regulation of transcription from RNA polymerase II promoter in response to glucose starvation [GO:0061406]; positive regulation of transcription from RNA polymerase II promoter in response to nitrogen starvation [GO:0036278]; protein localization to bud neck [GO:0097271]; regulation of cytoplasmic mRNA processing body assembly [GO:0010603]
	W0TG70	Proteinase YSCB	cellular response to starvation [GO:0009267]; pexophagy [GO:0000425]; protein catabolic process in the vacuole [GO:0007039]; sporulation resulting in formation of a cellular spore [GO:0030435]
Osmotic	W0TAV4	12 kDa heat shock protein	cell adhesion [GO:0007155]; cellular response to heat [GO:0034605]; cellular response to osmotic stress [GO:0071470]; cellular response to oxidative stress [GO:0034599]; plasma membrane organization [GO:0007009]
	W0T4K1	NAD(P)H-dependent D-xylose reductase	arabinose catabolic process [GO:0019568]; cellular response to osmotic stress [GO:0071470]; cellular response to oxidative stress [GO:0034599]; D-xylose catabolic process [GO:0042843]; galactose catabolic process [GO:0019388]
	W0TA21	Monothiol glutaredoxin-5	cellular response to oxidative stress [GO:0034599]; protein maturation by [2Fe-2S] cluster transfer [GO:0106034]; protein maturation by [4Fe-4S] cluster transfer [GO:0106035]; response to osmotic stress [GO:0006970]
Oxidative stress	W0TAV4	12 kDa heat shock protein	cell adhesion [GO:0007155]; cellular response to heat

			[GO:0034605]; cellular response to osmotic stress [GO:0071470]; cellular response to oxidative stress [GO:0034599]; plasma membrane organization [GO:0007009]
	W0TF05	Superoxide dismutase [Cu-Zn] (EC 1.15.1.1)	cellular copper ion homeostasis [GO:0006878]; cellular zinc ion homeostasis [GO:0006882]; fungal-type cell wall organization [GO:0031505]; negative regulation of cellular respiration [GO:1901856]; positive regulation of DNA-binding transcription factor activity [GO:0051091]; positive regulation of transcription from RNA polymerase II promoter in response to oxidative stress [GO:0036091]; protein stabilization [GO:0050821]
	W0T804	NADH-cytochrome b5 reductase (EC 1.6.2.2)	cellular response to oxidative stress [GO:0034599]; ergosterol biosynthetic process [GO:0006696]
	W0T4K1	NAD(P)H-dependent D-xylose reductase	arabinose catabolic process [GO:0019568]; cellular response to osmotic stress [GO:0071470]; cellular response to oxidative stress [GO:0034599]; D-xylose catabolic process [GO:0042843]; galactose catabolic process [GO:0019388]
	W0T992	Peroxiredoxin type-2	cell redox homeostasis [GO:0045454]; cellular response to oxidative stress [GO:0034599]; response to metal ion [GO:0010038]
	W0TFP1	Peroxidase (EC 1.11.1.-)	cellular response to oxidative stress [GO:0034599]
	W0TC44	Peroxiredoxin TSA1	cell redox homeostasis [GO:0045454]; cellular detoxification of hydrogen peroxide [GO:0061692]; hydrogen peroxide catabolic process [GO:0042744]; negative regulation of DNA-binding transcription factor activity [GO:0043433]; positive regulation of transcription from RNA polymerase II promoter in response to oxidative stress [GO:0036091]
	W0TKG9	Mitochondrial peroxiredoxin PRX1	cell redox homeostasis [GO:0045454]; cellular response to oxidative stress [GO:0034599]; regulation of hydrogen peroxide-induced cell death [GO:1903205]; response to cadmium ion [GO:0046686]
	W0TD03	Peptide-methionine (S)-S-oxide reductase (EC 1.8.4.11)	cellular response to oxidative stress [GO:0034599]
	W0TG01	Translationally-controlled tumor protein homolog	cellular response to oxidative stress [GO:0034599]; cytoplasmic translation [GO:0002181]; negative regulation of autophagy [GO:0010507]; negative regulation of microtubule depolymerization [GO:0007026]
	W0THB9	Carbonic anhydrase (EC 4.2.1.1) (Carbonate dehydratase)	carbon utilization [GO:0015976]; cellular response to carbon dioxide [GO:0071244]; cellular response to oxidative stress [GO:0034599]
	W0T7R5	Alcohol dehydrogenase (EC 1.1.1.1)	cellular response to oxidative stress [GO:0034599]
	W0TAN7	Glutamate decarboxylase (EC 4.1.1.15)	cellular response to oxidative stress [GO:0034599]; glutamate catabolic process [GO:0006538]
	W0TA21	Monothiol glutaredoxin-5	cellular response to oxidative stress [GO:0034599]; protein maturation by [2Fe-2S] cluster transfer [GO:0106034]; protein maturation by [4Fe-4S] cluster transfer [GO:0106035]; response to osmotic stress [GO:0006970]
	W0TFY8	Survival factor 1	cellular response to oxidative stress [GO:0034599]
	W0TF38	Succinate-semialdehyde dehydrogenase (EC 1.2.1.16)	cellular response to oxidative stress [GO:0034599]; gamma-aminobutyric acid catabolic process [GO:0009450]; glutamate decarboxylation to succinate [GO:0006540]
Protein folding	W0THY0	Heat shock protein SSA3	protein folding [GO:0006457]; SRP-dependent cotranslational protein targeting to membrane, translocation [GO:0006616]
	W0TCK6	Peptidyl-prolyl cis-trans isomerase (PPIase) (EC 5.2.1.8)	ascospore formation [GO:0030437]; histone deacetylation [GO:0016575]; positive regulation of meiotic nuclear division [GO:0045836]; protein folding [GO:0006457]
	W0TD70	ATP-dependent molecular chaperone HSC82	adenylate cyclase-activating glucose-activated G protein-coupled receptor signaling pathway [GO:0010619]; chaperone-mediated protein folding [GO:0061077]; heterochromatin assembly by small RNA [GO:0031048]
	W0T4B9	Protein disulfide-isomerase (EC 5.3.4.1)	mannose trimming involved in glycoprotein ERAD pathway [GO:1904382]; protein folding [GO:0006457]
	W0TAQ8	Heat shock protein 104	cellular heat acclimation [GO:0070370]; chaperone cofactor-dependent protein refolding [GO:0051085]; protein folding in endoplasmic reticulum [GO:0034975]; protein unfolding [GO:0043335]; stress granule disassembly [GO:0035617]; trehalose metabolism in response to heat stress [GO:0070414]
	W0TBY6	Peptidyl-prolyl cis-trans isomerase (PPIase) (EC 5.2.1.8)	apoptotic process [GO:0006915]; protein folding [GO:0006457]
	W0T5P3	Heat shock protein 60	'de novo' protein folding [GO:0006458]; chaperone-mediated protein complex assembly [GO:0051131]; protein import into mitochondrial intermembrane space [GO:0045041]; protein

			maturation [GO:0051604]; protein refolding [GO:0042026]; protein stabilization [GO:0050821]
	W0TCA0	Peptidyl-prolyl cis-trans isomerase (PPIase) (EC 5.2.1.8)	protein folding [GO:0006457]
	W0TCL3	Hsp90 co-chaperone AHA1	cellular response to heat [GO:0034605]; protein folding [GO:0006457]
	W0TH33	Peptidylprolyl isomerase (EC 5.2.1.8)	chromatin organization [GO:0006325]; protein folding [GO:0006457]; regulation of homoserine biosynthetic process [GO:1901710]
	W0TF66	Prefoldin subunit 4	cytoplasm protein quality control by the ubiquitin-proteasome system [GO:0071629]; positive regulation of transcription elongation from RNA polymerase II promoter [GO:0032968]; protein folding [GO:0006457]; tubulin complex assembly [GO:0007021]

Supplementary Table S4. The identified proteins from 288 hours lactate cultured *Kluyveromyces marxianus* medium by LC-MS/MS analysis.

Metabolism	UniProt accession number	Protein name	Gene Ontology (Biological process)
Tricarboxylic acid cycle	W0TEG9	Malate dehydrogenase (EC 1.1.1.37)	carbohydrate metabolic process [GO:0005975]; malate metabolic process [GO:0006108]; tricarboxylic acid cycle [GO:0006099]
	W0TAI6	Malate dehydrogenase (EC 1.1.1.37)	carboxylic acid metabolic process [GO:0019752]; gluconeogenesis [GO:0006094]; protein import into peroxisome matrix [GO:0016558]; tricarboxylic acid cycle [GO:0006099]
	W0TJM6	Fumarate hydratase (EC 4.2.1.2)	double-strand break repair [GO:0006302]; fumarate metabolic process [GO:0006106]; tricarboxylic acid cycle [GO:0006099]
	W0THE4	Malate synthase (EC 2.3.3.9)	glyoxylate cycle [GO:0006097]; tricarboxylic acid cycle [GO:0006099]
	W0TBI1	Isocitrate dehydrogenase [NADP] (EC 1.1.1.42)	isocitrate metabolic process [GO:0006102]; tricarboxylic acid cycle [GO:0006099]
	W0TGG7	Isocitrate dehydrogenase [NAD] subunit, mitochondrial	isocitrate metabolic process [GO:0006102]; tricarboxylic acid cycle [GO:0006099]
	W0T7K6	Aconitate hydratase, mitochondrial (Aconitase) (EC 4.2.1.-)	mitochondrial genome maintenance [GO:0000002]; tricarboxylic acid cycle [GO:0006099]
	W0TFA8	Citrate synthase	propionate catabolic process, 2-methylcitrate cycle [GO:0019629]; tricarboxylic acid cycle [GO:0006099]
	W0T883	Succinate--CoA ligase [ADP-forming] subunit alpha, mitochondrial (EC 6.2.1.5) (Succinyl-CoA synthetase subunit alpha) (SCS-alpha)	succinyl-CoA metabolic process [GO:0006104]; tricarboxylic acid cycle [GO:0006099]
	W0T4J0	Succinate--CoA ligase [ADP-forming] subunit beta, mitochondrial (EC 6.2.1.5) (Succinyl-CoA synthetase beta chain) (SCS-beta)	succinyl-CoA metabolic process [GO:0006104]; tricarboxylic acid cycle [GO:0006099]
	W0TKL8	Succinate dehydrogenase [ubiquinone] iron-sulfur subunit, mitochondrial (EC 1.3.5.1)	tricarboxylic acid cycle [GO:0006099]
	W0T7K3	Isocitrate dehydrogenase [NAD] subunit, mitochondrial	tricarboxylic acid cycle [GO:0006099]
	W0TDY2	2-oxoglutarate dehydrogenase E1 component	tricarboxylic acid cycle [GO:0006099]
Glycolysis/gluconeogenesis	W0TAI6	Malate dehydrogenase (EC 1.1.1.37)	carboxylic acid metabolic process [GO:0019752]; gluconeogenesis [GO:0006094]; protein import into peroxisome matrix [GO:0016558]; tricarboxylic acid cycle [GO:0006099]
	W0TF96	Fructose-bisphosphatase (EC 3.1.3.11)	cellular response to glucose starvation [GO:0042149]; gluconeogenesis [GO:0006094]; reactive oxygen species metabolic process [GO:0072593]
	W0TD31	Phosphoenolpyruvate carboxykinase (ATP) (EC 4.1.1.49)	gluconeogenesis [GO:0006094]
	W0T3B0	Pyruvate carboxylase (EC 6.4.1.1)	gluconeogenesis [GO:0006094]; pyruvate metabolic process [GO:0006090]
	W0T3P9	Phosphotransferase (EC 2.7.1.-)	cellular glucose homeostasis [GO:0001678]; fructose 6-phosphate metabolic process [GO:0006002]; glycolytic fermentation [GO:0019660]; glycolytic process [GO:0006096]; mannose metabolic process [GO:0006013]
	W0TF55	Phosphotransferase (EC 2.7.1.-)	cellular glucose homeostasis [GO:0001678]; glycolytic process [GO:0006096]
	W0T3H5	Phosphoglycerate kinase (EC 2.7.2.3)	gluconeogenesis [GO:0006094]; glycolytic process [GO:0006096]
	W0T9W3	Fructose-bisphosphate aldolase (FBP aldolase) (EC 4.1.2.13)	gluconeogenesis [GO:0006094]; glycolytic process [GO:0006096]
	W0T4R5	Glucose-6-phosphate isomerase (EC 5.3.1.9)	gluconeogenesis [GO:0006094]; glycolytic process [GO:0006096]

	P84998	Glyceraldehyde-3-phosphate dehydrogenase 1 (GAPDH 1) (EC 1.2.1.12)	glucose metabolic process [GO:0006006]; glycolytic process [GO:0006096]
	A4ZGQ9	Glyceraldehyde-3-phosphate dehydrogenase (EC 1.2.1.12)	glucose metabolic process [GO:0006006]; glycolytic process [GO:0006096]
	W0T7K9	Phosphopyruvate hydratase (EC 4.2.1.11)	glycolytic process [GO:0006096]
	W0T6W0	Phosphoglycerate mutase 3	glycolytic process [GO:0006096]
Pentose-phosphate pathway	W0TCV6	Transaldolase (EC 2.2.1.2)	carbohydrate metabolic process [GO:0005975]; pentose-phosphate shunt [GO:0006098]
	W0T4I6	6-phosphogluconolactonase-like protein	carbohydrate metabolic process [GO:0005975]; pentose-phosphate shunt [GO:0006098]
	W0TD99	Ribulose-phosphate 3-epimerase (EC 5.1.3.1)	carbohydrate metabolic process [GO:0005975]; pentose-phosphate shunt [GO:0006098]
	W0T3V9	6-phosphogluconate dehydrogenase, decarboxylating (EC 1.1.1.44)	D-gluconate metabolic process [GO:0019521]; pentose-phosphate shunt [GO:0006098]
	W0TCY6	Glucose-6-phosphate 1-dehydrogenase (EC 1.1.1.49)	glucose metabolic process [GO:0006006]; pentose-phosphate shunt [GO:0006098]
Galactose metabolism	W0T4K1	NAD(P)H-dependent D-xylose reductase	arabinose catabolic process [GO:0019568]; cellular response to osmotic stress [GO:0071470]; cellular response to oxidative stress [GO:0034599]; D-xylose catabolic process [GO:0042843]; galactose catabolic process [GO:0019388]
	W0T9M7	Galactose-1-phosphate uridylyltransferase (EC 2.7.7.12)	galactose catabolic process via UDP-galactose [GO:0033499]
	S5WA50	UDP-glucose 4-epimerase	galactose metabolic process [GO:0006012]
	S5VNV2	Galactokinase (EC 2.7.1.6) (Galactose kinase)	galactose metabolic process [GO:0006012]
Other carbohydrate metabolism	W0TBD3	Phosphoglucomutase-2	carbohydrate metabolic process [GO:0005975]
	W0TDC6	Uncharacterized glycosyl hydrolase YBR056W	carbohydrate metabolic process [GO:0005975]
	W0TK22	Probable family 17 glucosidase SCW4	carbohydrate metabolic process [GO:0005975]
	B3GQU5	Exo-inulinase (EC 3.2.1.7) (Inulinase)	carbohydrate metabolic process [GO:0005975]
	W0TC08	Glycosidase (EC 3.2.-.-)	carbohydrate metabolic process [GO:0005975]; cell wall chitin metabolic process [GO:0006037]; fungal-type cell wall organization [GO:0031505]
	W0T4T5	Glycerol-3-phosphate dehydrogenase [NAD(+)] (EC 1.1.1.8)	carbohydrate metabolic process [GO:0005975]; glycerol-3-phosphate catabolic process [GO:0046168]
Lactate metabolism	W0TFC1	D-lactate dehydrogenase [cytochrome] 2	lactate catabolic process [GO:1903457]
	W0T5A6	Cytochrome b2	lactate metabolic process [GO:0006089]
**Stress response**			
Starvation	W0T855	cAMP-dependent protein kinase regulatory subunit	cAMP-mediated signaling [GO:0019933]; negative regulation of meiotic cell cycle [GO:0051447]; negative regulation of Ras protein signal transduction [GO:0046580]; positive regulation of adenylate cyclase activity [GO:0045762]; positive regulation of protein export from nucleus [GO:0046827]; positive regulation of transcription from RNA polymerase II promoter in response to glucose starvation [GO:0061406]; positive regulation of transcription from RNA polymerase II promoter in response to nitrogen starvation [GO:0036278]; protein localization to bud neck [GO:0097271]; regulation of cytoplasmic mRNA processing body assembly [GO:0010603]
	W0TF96	Fructose-bisphosphatase (EC 3.1.3.11)	cellular response to glucose starvation [GO:0042149]; gluconeogenesis [GO:0006094]; reactive oxygen species metabolic process [GO:0072593]
	W0T2R7	Protein SDS23	cellular response to glucose starvation [GO:0042149]; regulation of mitotic metaphase/anaphase transition [GO:0030071]

	W0TCL5	Zinc finger protein ZPR1	cellular response to starvation [GO:0009267]; G2/M transition of mitotic cell cycle [GO:0000086]; response to glucose [GO:0009749]
	W0TG70	Proteinase YSCB	cellular response to starvation [GO:0009267]; pexophagy [GO:0000425]; protein catabolic process in the vacuole [GO:0007039]; sporulation resulting in formation of a cellular spore [GO:0030435]
Osmotic	W0T4K1	NAD(P)H-dependent D-xylose reductase	arabinose catabolic process [GO:0019568]; cellular response to osmotic stress [GO:0071470]; cellular response to oxidative stress [GO:0034599]; D-xylose catabolic process [GO:0042843]; galactose catabolic process [GO:0019388]
	W0T9D0	Serine/threonine-protein phosphatase 2A activator (EC 5.2.1.8) (Phosphotyrosyl phosphatase activator)	autophagy [GO:0006914]; DNA repair [GO:0006281]; G1/S transition of mitotic cell cycle [GO:0000082]; mitotic spindle organization [GO:0007052]; regulation of transcription from RNA polymerase II promoter in response to stress [GO:0043618]; response to osmotic stress [GO:0006970]
	W0TAV4	12 kDa heat shock protein	cell adhesion [GO:0007155]; cellular response to heat [GO:0034605]; cellular response to osmotic stress [GO:0071470]; cellular response to oxidative stress [GO:0034599]; plasma membrane organization [GO:0007009]
	W0TGP2	Glutamine synthetase (EC 6.3.1.2)	cellular response to osmotic stress [GO:0071470]; glutamine biosynthetic process [GO:0006542]
	W0TDZ5	Clustered mitochondria protein homolog (Protein TIF31 homolog)	cellular response to osmotic stress [GO:0071470]; intracellular distribution of mitochondria [GO:0048312]
	W0T7Z4	Ankyrin repeat-containing protein YAR1	cellular response to oxidative stress [GO:0034599]; regulation of protein localization [GO:0032880]; response to osmotic stress [GO:0006970]; ribosomal small subunit biogenesis [GO:0042274]; ribosomal small subunit export from nucleus [GO:0000056]
	W0T9K6	Nuclear cap-binding protein complex subunit 1	mRNA cis splicing, via spliceosome [GO:0045292]; mRNA transport [GO:0051028]; nuclear-transcribed mRNA catabolic process, nonsense-mediated decay [GO:0000184]; response to osmotic stress [GO:0006970]
	W0TAQ8	Heat shock protein 104	cellular heat acclimation [GO:0070370]; cellular response to osmotic stress [GO:0071470]; chaperone cofactor-dependent protein refolding [GO:0051085]; inheritance of oxidatively modified proteins involved in replicative cell aging [GO:0001319]; protein folding in endoplasmic reticulum [GO:0034975]; protein unfolding [GO:0043335]; stress granule disassembly [GO:0035617]; trehalose metabolism in response to heat stress [GO:0070414]
Oxidative stress	W0TF05	Superoxide dismutase [Cu-Zn] (EC 1.15.1.1)	age-dependent response to reactive oxygen species involved in chronological cell aging [GO:0001320]; cellular copper ion homeostasis [GO:0006878]; cellular zinc ion homeostasis [GO:0006882]; fungal-type cell wall organization [GO:0031505]; negative regulation of cellular respiration [GO:1901856]; positive regulation of DNA-binding transcription factor activity [GO:0051091]; positive regulation of transcription from RNA polymerase II promoter in response to oxidative stress [GO:0036091]; protein stabilization [GO:0050821]
	W0T4K1	NAD(P)H-dependent D-xylose reductase	arabinose catabolic process [GO:0019568]; cellular response to osmotic stress [GO:0071470]; cellular response to oxidative stress [GO:0034599]; D-xylose catabolic process [GO:0042843]; galactose catabolic process [GO:0019388]
	W0TIE1	Actin	ascospore wall assembly [GO:0030476]; cellular response to oxidative stress [GO:0034599]; DNA repair [GO:0006281]; endocytosis [GO:0006897]; establishment of cell polarity [GO:0030010]; mitotic actomyosin contractile ring contraction [GO:1902404]; protein secretion [GO:0009306]; vacuole inheritance [GO:0000011]
	W0TAV4	12 kDa heat shock protein	cell adhesion [GO:0007155]; cellular response to heat [GO:0034605]; cellular response to osmotic stress [GO:0071470]; cellular response to oxidative stress [GO:0034599]; plasma membrane organization [GO:0007009]
	W0TC44	Peroxiredoxin TSA1	cell redox homeostasis [GO:0045454]; cellular detoxification of hydrogen peroxide [GO:0061692]; hydrogen peroxide catabolic process [GO:0042744]; negative regulation of DNA-binding transcription factor activity [GO:0043433]; positive regulation of transcription from RNA polymerase II promoter in response to oxidative stress [GO:0036091]
	W0T8K0	Glutathione reductase (EC 1.8.1.7)	cell redox homeostasis [GO:0045454]; cellular response to menadione [GO:0036245]; cellular response to oxidative stress [GO:0034599]; glutathione metabolic process [GO:0006749]; protein glutathionylation [GO:0010731]

	W0TCC1	Peroxiredoxin DOT5	cell redox homeostasis [GO:0045454]; cellular response to oxidative stress [GO:0034599]
	W0TKG9	Mitochondrial peroxiredoxin PRX1	cell redox homeostasis [GO:0045454]; cellular response to oxidative stress [GO:0034599]; regulation of hydrogen peroxide-induced cell death [GO:1903205]; response to cadmium ion [GO:0046686]
	W0TFP1	Peroxidase (EC 1.11.1.-)	cellular response to oxidative stress [GO:0034599]
	W0T647	Glutathione peroxidase	cellular response to oxidative stress [GO:0034599]
	W0T7R5	Alcohol dehydrogenase (EC 1.1.1.1)	cellular response to oxidative stress [GO:0034599]
	W0TFY8	Survival factor 1	cellular response to oxidative stress [GO:0034599]
	W0TG01	Translationally-controlled tumor protein homolog	cellular response to oxidative stress [GO:0034599]; cytoplasmic translation [GO:0002181]; negative regulation of autophagy [GO:0010507]; negative regulation of microtubule depolymerization [GO:0007026]
	W0T804	NADH-cytochrome b5 reductase (EC 1.6.2.2)	cellular response to oxidative stress [GO:0034599]; ergosterol biosynthetic process [GO:0006696]
	W0TAN7	Glutamate decarboxylase (EC 4.1.1.15)	cellular response to oxidative stress [GO:0034599]; glutamate catabolic process [GO:0006538]
	W0T7Z4	Ankyrin repeat-containing protein YAR1	cellular response to oxidative stress [GO:0034599]; regulation of protein localization [GO:0032880]; response to osmotic stress [GO:0006970]; ribosomal small subunit biogenesis [GO:0042274]; ribosomal small subunit export from nucleus [GO:0000056]
	W0T3L1	RNA polymerase II subunit A C-terminal domain phosphatase (EC 3.1.3.16)	dephosphorylation of RNA polymerase II C-terminal domain [GO:0070940]; response to oxidative stress [GO:0006979]
Protein folding	W0TD70	ATP-dependent molecular chaperone HSC82	adenylate cyclase-activating glucose-activated G protein-coupled receptor signaling pathway [GO:0010619]; chaperone-mediated protein folding [GO:0061077]; heterochromatin assembly by small RNA [GO:0031048]
	W0TBY6	Peptidyl-prolyl cis-trans isomerase (PPIase) (EC 5.2.1.8)	apoptotic process [GO:0006915]; protein folding [GO:0006457]
	W0TCK6	Peptidyl-prolyl cis-trans isomerase (PPIase) (EC 5.2.1.8)	ascospore formation [GO:0030437]; histone deacetylation [GO:0016575]; positive regulation of meiotic nuclear division [GO:0045836]; protein folding [GO:0006457]
	W0T7S8	ATPase GET3 (EC 3.6.-.-) (Arsenical pump-driving ATPase) (Arsenite-stimulated ATPase) (Golgi to ER traffic protein 3) (Guided entry of tail-anchored proteins 3)	ATP-independent chaperone mediated protein folding [GO:1990507]; pheromone-dependent signal transduction involved in conjugation with cellular fusion [GO:0000750]; posttranslational protein targeting to endoplasmic reticulum membrane [GO:0006620]; protein insertion into ER membrane [GO:0045048]; response to heat [GO:0009408]; response to metal ion [GO:0010038]; retrograde vesicle-mediated transport, Golgi to endoplasmic reticulum [GO:0006890]
	W0T4W1	Heat shock protein homolog SSE1	autophagy [GO:0006914]; proteasomal ubiquitin-independent protein catabolic process [GO:0010499]; proteasome-mediated ubiquitin-dependent protein catabolic process [GO:0043161]; protein refolding [GO:0042026]
	W0T4B9	Protein disulfide-isomerase (EC 5.3.4.1)	cell redox homeostasis [GO:0045454]; mannose trimming involved in glycoprotein ERAD pathway [GO:1904382]; protein folding [GO:0006457]
	W0TAQ8	Heat shock protein 104	cellular heat acclimation [GO:0070370]; cellular response to osmotic stress [GO:0071470]; chaperone cofactor-dependent protein refolding [GO:0051085]; inheritance of oxidatively modified proteins involved in replicative cell aging [GO:0001319]; protein folding in endoplasmic reticulum [GO:0034975]; protein unfolding [GO:0043335]; stress granule disassembly [GO:0035617]; trehalose metabolism in response to heat stress [GO:0070414]
	W0TCL3	Hsp90 co-chaperone AHA1	cellular response to heat [GO:0034605]; protein folding [GO:0006457]
	W0THY0	Heat shock protein SSA3	cellular response to heat [GO:0034605]; protein folding [GO:0006457]; SRP-dependent cotranslational protein targeting to membrane, translocation [GO:0006616]
	W0TIR2	Putative aspartyl aminopeptidase	chaperone-mediated protein folding [GO:0061077]
	W0TH33	Peptidylprolyl isomerase (EC 5.2.1.8)	chromatin organization [GO:0006325]; protein folding [GO:0006457]; regulation of homoserine biosynthetic process [GO:1901710]
	W0T5P3	Heat shock protein 60	'de novo' protein folding [GO:0006458]; chaperone-mediated protein complex assembly [GO:0051131]; protein import into mitochondrial intermembrane space [GO:0045041]; protein

			maturation [GO:0051604]; protein refolding [GO:0042026]; protein stabilization [GO:0050821]
	W0TDW0	Peptidyl-prolyl cis-trans isomerase D	protein refolding [GO:0042026]

Supplementary Table S5. All primer sequences for multilocus sequence typing and qPCR analysis.

Gene	Forward primer	Reverse primer
**For multilocus sequence typing**		
IPP1	ATCGGTGCCAAGAACACCTT	TTGTCGATTGGCTCGTCTGG
TFC1	AAGGCCGATTTGGGTCAAAC	TCTGCGGACTCAGAGTTATGC
GPH1	TGGAACACTGTGAAGCAGCA	TTTCGTCAGCGTACTCCTGG
GSY2	CACGCCATGAGATTCCCTCA	CGTCCTCTTCGTCGTCATCC
SGA1	CTCCGATGGTTCGGGTCAAT	TCATGGGTCAAGGTACTGGC
**For quantitative PCR**		
HSP12	CCAGACTCCCAAAAGAGCACTC	CCTTACCCTGTTGAGCACTGTC
GAP1	CCAAGAAAAGGACCCAGCTACC	GGTCTTGGAAGGAGTAATC
ENO	GGTAAGGGTGTCTTGAAGGCTG	AGCACCCAACTTGGACTTGTTT
PGU1	GTCGGGCAGGTTACCTTTGATT	CGACATCTGAGTTGCCAGTCAA
INU1	AACACTCCAAACGAGAAGGACG	CGGAGTTAGGGTTTGTGCTGAA
ACT	GGGGCTTCGGTCAACAAAAC	TGGTCGGTATGGGTCAAAAGG

The uncropped gel in Figure 2b. The image was taken by an iPhone 6 (Camera app in iOS 12.5.1, Apple).

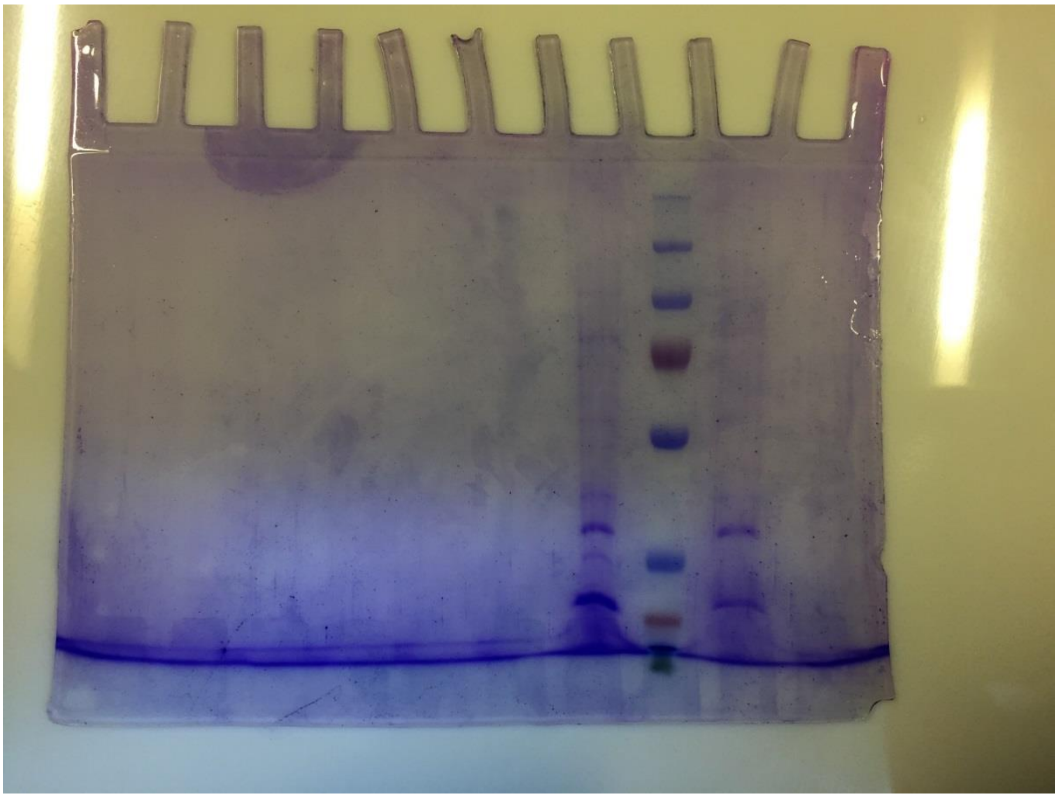

The uncropped gel in Figure S3.

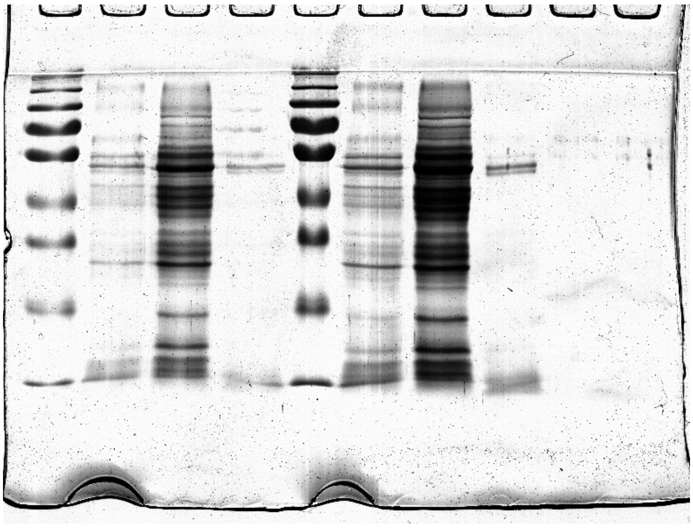

Supplement: Supplementary file 1 — Supplementary Information 1. [file 41598_2021_94101_MOESM1_ESM.pdf]
